# Are redox catalytic reaction rates accelerated in microdroplets on electrode surfaces?

**DOI:** 10.1007/s10008-025-06283-4

**Published:** 2025-04-12

**Authors:** Nathan S. Lawrence, Jay D. Wadhawan

**Affiliations:** School of Engineering, Chemical Engineering, The University of Hull, Cottingham Road, Kingston-Upon-Hull, HU6 7RX UK

**Keywords:** Microdroplets, Three-phase boundary, Redox catalysis, Reaction acceleration, Droplet voltammetry, Triple phase junction

## Abstract

Homogeneous redox catalysis within electrochemically supported microdroplets immobilised on an electrode surface and bathed by an immiscible electrolyte solution is characterised using finite difference numerical methods, after conformal transformation of the physical problem. This is shown to be a challenging environment to simulate and model, not least due to the confinement of the heterogeneous electron transfer to the droplet/support/electrolyte boundary, and hence leading to acute convergent/divergent diffusion regimes. Reactivity at the triple phase boundary underpins both the spatial and temporal non-uniformity of the reacting droplet environment. Crucially, through comparison with experimental data reported in the literature, it is demonstrated that *there is no droplet-induced acceleration of the redox catalytic reaction.* Reasons for this discrepancy with literature are suggested. It is recommended that any inference of reaction rate acceleration through increased rate constants in microdroplets on surfaces be re-examined, lest the multi-dimensional dynamics at the three-phase boundary are unaccounted.

**Supplementary Information:**

The online version contains supplementary material available at 10.1007/s10008-025-06283-4.

## Background

Cellular compartmentalisation causes a plethora of important biological chemical reactions to take place within confined and spatially organised systems [1]. Microdroplet reactors (liquid reactors of, at most, microlitre volumes) have been considered to act as crude mimics to these systems, and the ease by which they may be created either through droplet ensembles [2] or as single entities [3] has encouraged a flurry of activity in analytical and industrial chemistry over the last 25 years [4–6]. Interleaved with advances in micro-fabrication techniques and methods to enable single microdroplets to be probed has been the increasing recognition of the functional importance played by protein-rich droplets (biomolecular condensates or membraneless compartments, stress granules, P-bodies and the nucleolus) in biological processes, such as their wetting on membranes for intracellular organisation [7, 8], and in driving spontaneous redox reactions in living cells [9]. Concurrently, the exciting and remarkable discovery that a variety of reactions within microdroplets exhibit accelerated reaction rates resulting from increased rate constants, compared with bulk solution [10], has led to a plethora of mechanistic studies designed to unravel the physicochemical rationales underpinning such phenomena [11–14], and the creative exploration of the limits of what might be achieved.

Droplet environments are confined, volumetric spaces. For spherical droplets, the volume is proportional to the cube of the radius, so that millimetric droplets are micro/nanolitre reactors; micrometric droplets have volumes on the order of several femtolitres, and nanometric droplets, such as those generated using liquid nanotechnology, can [15] occupy a few yoctolitres. Since the hydrated proton in water is thought to occupy a volume of approximately 1.1 yL in the bulk state [16], the possibility of partially solvated species (“heterogeneous water”) in nanometric space can account for unusual reactivity [17–19]. Additionally, there is an interplay between surface and bulk reactivity [20]—species close to, or at, the fluid/fluid interface, which marks the boundary between a liquid droplet and its surrounding liquid or gaseous bathing environment, can give rise to heterogeneous or interfacial reactivity that is markedly different to that which occurs homogeneously in bulk solution [21–23]. Furthermore, the influence of perturbations of electrical, magnetic, gravitational or acoustic fields in the environment bathing the droplet may also lead to alterations in reactivity within droplets [24].

For droplets confined to surfaces and bathed by fluids, the droplet geometry is impacted by the line tension [25]—the energy per unit length of the three-phase boundary that forms the base circumference of the droplet—where the solid electrode meets both the liquid droplet and the bathing fluid [26, 27], and its Eötvös number (Bond number)—the ratio of gravitational forces to surface tension. For droplets of mass less than *ca.* 1 mg [28], gravitational and buoyancy forces can be neglected, so that the droplets take up the shape of a sessile, spherical cap; larger droplet masses tend to exhibit distortion, either through a flattening of the surface [29] or *via* buoyant uplift to afford pear-shaped deposits, as has been visualised for the case of large dodecane droplets on surfaces bathed by a denser fluid [30].

Electrochemistry within droplets, where the surface is an electrode, has been examined for the last half-century, pioneered by Marken, where droplets were either generated in the bulk of a solution using acoustic emulsification of a liquid/liquid interface and transported to an electrode surface [6, 31–35], or directly formed on an electrode surface through solvent evaporation of a liquid material contained in an aliquot of volatile solvent pipetted onto the electrode [6, 20, 35–47]. The latter type of experiment involving droplet-modified electrodes was inspired by work developed by Scholz on particle and droplet electrochemistry [48, 49] and the biphasic droplet systems developed by Wendt [50] and Nakatani [51]. In such experiments, the external electric field is exquisitely controlled by the potentiostatic instrumentation, enabling both Faradaic reactivity and electrowetting [52] to be modulated. However, mass transport to the electrode surface (both inside and outside of the droplet) is not uniform, owing to the occurrence of a singularity at the triple phase junction. This is also the case when the droplets contain sufficient supporting electrolyte so that heterogeneous electron transfer can take place across the basal contacting plane between the sessile droplet and the electrode [53]. Accordingly, the quantitative inference of homogeneous rate constants resulting from reactivity following heterogeneous electron transfer at the solid surface needs to account for the droplet-confined, acute convergent/divergent transport regime at the triple phase boundary, which is akin to the familiar “edge effect” at microelectrodes [54]. Whilst this is notoriously difficult to model, methods to simulate diffusive [26, 27, 29, 30, 50, 51, 53, 55–62] and convective [63] mass transport have, nevertheless, been developed using finite difference, finite element and boundary element methods. These have indicated that numerical simulation techniques require a very dense mesh/number of nodes close to the singularity, which causes lengthy computations, and due care and attention must be given to ensuring accurate convergence of the numerical simulation.

In recent work [64–67], redox catalytic rate constants inside electroactive droplets have been quantified either from thin film chronoamperometry or through comparison of experimental data with numerical simulations developed using a commercial finite element package. Surprisingly, these have demonstrated that even in micrometric and millimetric droplet environments (where the volume of the droplets is up to 18 orders of magnitude larger than the volume occupied by a single, fully hydrated proton), larger *rate constants* are observed compared with those measured under semi-infinite conditions [65], and that, for enzyme reactions, the homogeneous rate constant increases as droplet size decreases [64, 67]. Such rate constant measurements have relied on their extraction from experimental voltammetric data through numerical simulation. Given that electrochemically mediated homogeneous (photo)redox reactions (EC’ processes) are extensively employed for a sustainable industrial future (such as in solar fuel cells, carbon dioxide conversion and wastewater remediation) [68–70], in point-of-practice healthcare technologies (such as blood glucose measurement) [71–73], and in understanding the redox chemistry within compartmentalised biology, the occurrence of reaction rate acceleration is of major significance. A key question underpinning the inference of reaction acceleration in droplet environments is whether the models used are suitable for the highly challenging situations where a triple phase boundary exists.

In this work, we seek to address this question through exploring the classical mechanism for electrochemically induced redox catalysis (EC’ reaction) within single, supported, electroactive oil droplets immobilised on an electrode surface and bathed by an inert aqueous electrolyte, so as to explore the impact that both homogeneous reaction kinetics and droplet contact angle have on diffusion-only voltammetry with these droplets, and how these compare with semi-infinite, one-dimensional systems, *viz*. those in which planar diffusion regimes occur, and, where relevant, when there is exhaustive consumption of the substrate in a very thin layer. In the Supporting Information (SI), we provide an overview of the EC’ reaction through the examination of the voltammetric relationships through their kinetic zones (S1), paying particular attention to the zones important for the design and development of electrochemical manufacture processes and (bio)sensors. We then (S2-S5) outline the models employed in this work, using the seminal conformal transformation developed by Amatore et al. [61]. We focus the remainder of this paper on the discussion of the results from five kinetic zones and subsequently examine experimental data, reported in the literature [65], pertaining to the droplet version of a mechanism we have previously studied at both planar electrodes and at droplet-modified electrodes, viz. the oxidation of sulphydryl species by an electro-generated oxidant [74–85]. We demonstrate, unequivocally, that *no droplet-induced reaction rate acceleration takes place* in aqueous, microlitre droplets of ferrocyanide containing l-cysteine immobilised on a glassy carbon electrode and bathed by a 1,2-dichloroethane electrolyte.

## Results and discussion

We first examine the voltammetry corresponding to the EC’ reaction occurring within the confined environment of an electrochemically supported spherical cap droplet (of basal radius *a* = 0.5 mm) immobilised on an electrode surface and bathed by a fluid containing reference and counter electrodes. The contact angle, *θ*, of the droplet is set to one of three cases *θ* = ¼π, ½π or ¾π, so that we consider the effects of small droplets with acute convergent/divergent diffusion regimes (*θ* = ¼π), larger hemispherical droplets (*θ* = ½π), and even larger, partially non-wetting droplets (*θ* = ¾π). The droplets are sufficiently small and are electrochemically supported, so that transport within the droplets is considered to be only through diffusion (see S2).

As outlined in S1 and S2, the EC’ mechanism is:

1 $$\begin{array}{c}P-{e}^{-}\rightleftarrows Q\\ Q+M\stackrel{{k}_{EC^{\prime}}}{\to }P+Products\end{array}$$

where *P* is the reduced form of the mediator, *Q* is the oxidised form and *M* is the substrate (analyte). We assume that ion transfers across the droplet/fluid interface, resulting from the conservation of electroneutrality within the droplet, are rapid. Thus, the only heterogeneous reaction that is of interest is the electrode process for the inter-conversion of the mediator species, which occurs with formal potential *E*^0′^, and Bulter-Volmer kinetics (parameterised by the standard heterogeneous rate constant, *k*_s_, and the transfer coefficient, *α*). In this work, we set *α* = ½. Additionally, for the first part, we assume that all species have equal diffusion coefficients set at *D*_P_ = *D*_Q_ = *D*_M_ = 10^−5^ cm^2^ s^−1^. The only homogeneous reaction is that for redox catalysis, and we consider the rate constant for this reaction (*k*_EC′_) to take up one of two values: one close to the diffusion limit, *k*_EC′_ = 10^9^ M^−1^ s^−1^, or one typical for the mediated reaction of hydrogen sulphide or sulphydryl thiols in aqueous solution at neutral pH [74–85], *viz*. *k*_EC′_ = 10^3^ M^−1^ s^−1^. This enables the Damköhler number (the kinetic factor that is the ratio of the voltammetric timescale to the homogeneous reaction timescale), $\lambda =\frac{RT}{Fv}{k}_{Ec^{\prime}}{c}_{P}^{0}$, in which *R* is the molar gas constant, *T* is the absolute temperature, *F* is the Faraday constant, *v* is the voltammetric sweep rate and *c*_P_^0^ is the bulk solution concentration of the reduced form of the mediator, to vary over 12 orders of magnitude. As indicated in S2, the dimensionless scan rate, *s*^2^, is the inverse of the Fourier number, with $s=\frac{a}{\sqrt{{D}_{P}}}\sqrt{\frac{Fv}{RT}}$, which is allowed to vary over the range 10^0^ ≤ s ≤ 10^3^. The excess factor—the ratio of the bulk concentrations of the substrate (*M*) to the reduced form of the mediator (*P*), $\gamma =\frac{{C}_{M}^{0}}{{C}_{P}^{0}}$, takes one of two values: *γ* = 10^0^ or 10^3^. This curtails the kinetic zones that are considered herein, as explained in S1 and S2.

**Fig. 1:**
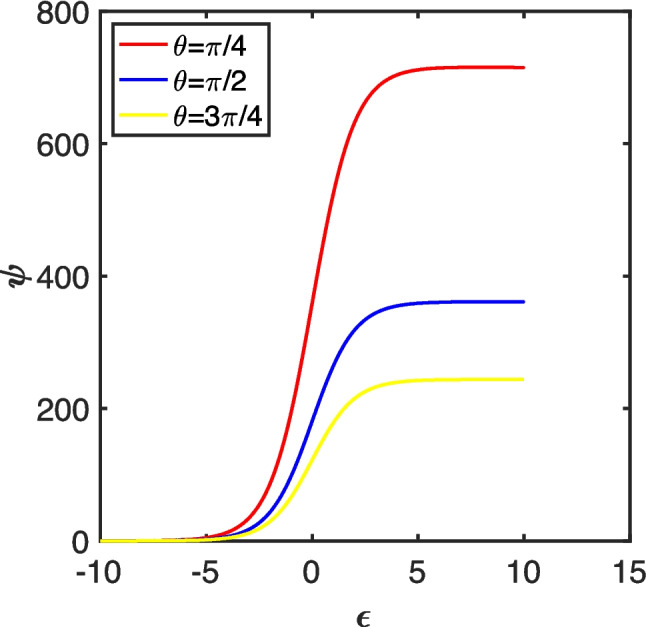
Quasi-steady-state LSVs corresponding to the electrochemically reversible oxidation of the mediator in the absence of the substrate, with *s* = 10.^6^, at *θ* = ¼π, ½π or ¾π (see key). Data reproduced from reference [53]

We assume further that the electrode kinetics for the one-electron transformation of the mediator couple are fast *vis-à-vis* the mass transport, so that voltammograms are generated under conditions where the Nernst equilibrium law dictates the concentrations of the two mediator forms at the electrode surface. This requires that the average mass transfer coefficient, $\overline{{k }_{L}}$, to be smaller than the standard heterogeneous rate constant, *viz*. $\overline{{k }_{L}}<{k}_{s}$ The mass transfer coefficient can be estimated from the transport-limiting current (*i*_lim_) through the relationship ${i}_{lim}=FS{c}_{P}^{0}\overline{{k }_{L}}$, where *S* is the electrode area exposed to the electroactive species, *i.e*. for a droplet, $S=\pi {a}^{2}$. As illustrated in previous work [53], for droplets, quasi-steady-state voltammograms are only observed when cylindrical diffusion dominates the transport regime, which occurs when *s* ~ 10^6^, see Fig. 1. In these cases, recognising that the limiting dimensionless current, *ψ*_lim_, is given by ${\psi }_{lim}=\frac{{i}_{lim}}{2\pi {FD}_{P}{ac}_{P}^{0}}$, the condition for electrochemical reversibility of a redox couple confined within a droplet is:

2 $${2\psi}_{\mathit l\mathit i\mathit m}\frac{D_P}a<k_s$$

Thus, systems comprising smaller droplets and/or larger diffusion coefficients tend towards electron transfer-limited behaviour. As illustrated in Fig. 1, the dimensionless limiting current is a function of the contact angle—for the three cases considered, the inverse power law, ${\psi }_{lim}=5.64\times {10}^{2}{\theta }^{-0.980}$, holds, with a coefficient of determination, *R*^2^ > 0.999. Clearly, the greatest mass transfer rate occurs for the most partially wetting droplet. This is expected since that case affords the most acute convergent/divergent diffusion regime. For the droplets modelled in the first part of this work, *D*_p_ = 10^−5^ cm^2^ s^−1^ with *a* = 0.5 mm, so that electrochemical reversibility is expected provided the heterogeneous rate constant is larger than 0.29 cm s^−1^ (for *θ* = ¼π), 0.14 cm s^−1^ (for *θ* = ½π) or 9.8 × 10^−2^ cm s^−1^ (for *θ* = ¾π). Thus, setting *k*_s_ = 3.1 cm s^−1^ in the simulations described below satisfies this condition for both the droplet scenarios and for those involving semi-infinite diffusion, since the Matsuda-Ayabe parameter [86], ${\Lambda }_{MA}=\frac{{k}_{s}}{\sqrt{{D}_{P}\frac{Fv}{RT}}}=\frac{{k}_{s}a}{s{D}_{P}}>15$ for all scan rates considered.

Thus, we first examine the case when the excess factor is unity, enabling an exploration of the split-wave total catalysis (KT2) and general kinetics (KG*) zones [87], and subsequently examine that when the substrate concentration is 1000 times that of the mediator: the pure kinetics (K), the no substrate consumption (KS) and kinetics diffusion (KD) zones [87]. In order that we can generalise the results, we report potentials (*E*), currents (*i*) and concentrations (*c*_J_) in dimensionless form, *viz* $\varepsilon =\frac{F}{RT}\left(E-{E}^{{0}^{\prime}}\right);\;\psi =\frac{i}{2\pi {FD}_{P}{ac}_{P}^{0}};\;j=\frac{{c}_{j}}{{c}_{P}^{0}}$.

### The total catalysis (KT) and general kinetics (KG*) zones

**Fig. 2:**
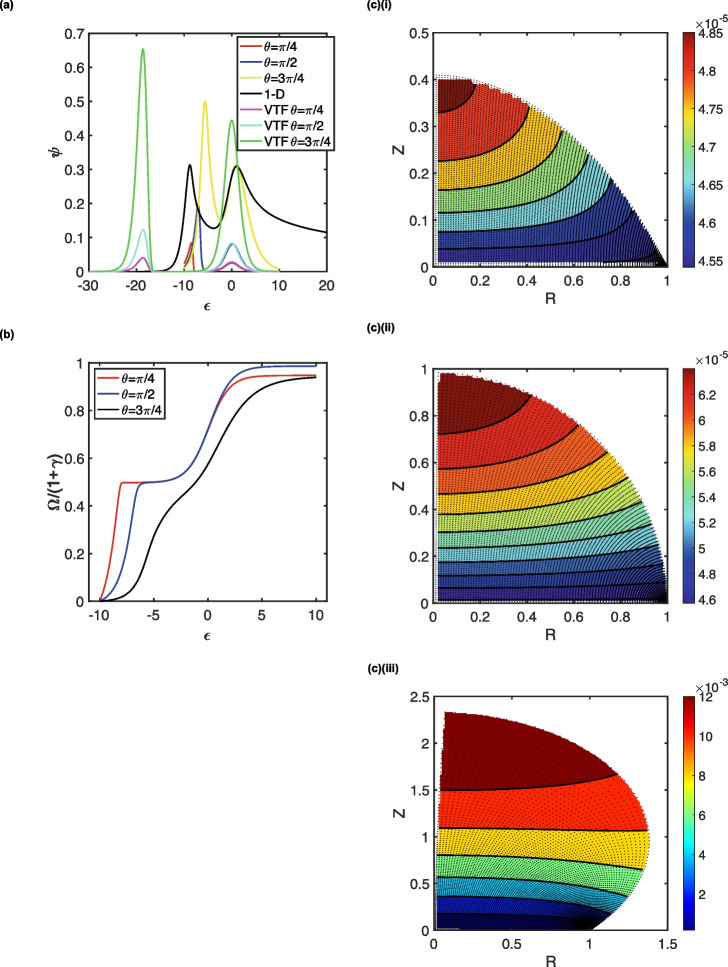
The KT2 zone of the EC’ mechanism in droplets (*s* = 1, *γ* = 1, *λ* = 1.25 × 10^8^). **a** LSVs corresponding to planar diffusion (black traces); very thin film electrolysis (VTF) or droplet diffusion at *θ* = ¼π, ½π or ¾π (see key). **b** Cumulative plots of the normalised integrated charge for the droplet case (see key). **c** Concentration profiles illustrating the non-uniformity of the extent by which the reduced form of the mediator (*P*) is depleted at the end of the LSV (*ε* = 10) for droplets of *θ* = ¼π (i), ½π (ii) and ¾π (iii)

For semi-infinite, planar diffusion, the kinetic zone diagram [87] for fast electrode kinetics and equal diffusion coefficients indicates that, when the excess factor is unity (the substrate and mediator concentrations are identical), the voltammetric waveshape changes from the KT2 (total catalysis with two peaks) to the KG* (general kinetics) zone when the Damköhler number (*λ*) becomes smaller than ~ 100. For the droplet case, when the size of the droplet is comparable with that of the planar depletion region, *viz* $a\sim\sqrt{D_P\frac{RT}{Fv}}$, the dimensionless scan rate, *s*^2^, is on the order of unity. This corresponds to an exhaustive electrolysis scenario [53]. Figure 2 depicts this case for reversible electrode kinetics (*k*_s_ = 3.1 cm s^−1^) where *s* = 1 and *γ* = 1, with *λ* corresponding to 1.25 × 10^8^. This unusually large value of the kinetic parameter corresponds to a homogeneous redox catalytic reaction that approaches the diffusion limit, *k*_EC′_ = 1.0 × 10^9^ M^−1^ s^−1^ for a droplet of radius *a* = 0.5 mm with all species having diffusion coefficients *D*_P_ = 1.0 × 10^−5^ cm^2^ s^−1^. The normalised cumulative fraction of the charge passed per equivalent of the mediator catalyst, $\frac{\Omega }{1+\gamma }$, where *Ω* is the relative amount of charge transferred to that corresponding to exhaustive consumption of the mediator is shown in Fig. 2b. Whilst this exhibits the expected trend of two waves, the simulations are accurate for the first wave but are > 90% converged for the second wave. The linear sweep voltammograms (LSVs) presented in Fig. 2a correspond to droplets of the same basal radius (*a*), with different contact angles (*θ* = ¼π, ½π and ¾π: red, blue and yellow traces), so that the droplet volume increases with contact angle. Also illustrated in Fig. 2a are the corresponding LSVs for the planar diffusion (black traces, see S1 and S4) case and for the very thin film scenario (see S5; magenta, cyan and green traces). These data exhibit several features. First, in all cases, two peaks are observed as expected for the KT2 region: the first corresponds to the mediated, irreversible oxidation of the substrate *M*, which takes place at a potential less negative of the *P*/*Q* redox potential (*ε* = 0), with the second, close to *ε* = 0, corresponding to the oxidation of the regenerated reduced form of the mediator at the electrode surface. In all cases, the first waveshape is skewed, as expected: the homogeneous reaction is so fast that it is controlled by diffusion of the substrate (*M*). In contrast, the second exhibits Gaussian characteristics, as anticipated for the exhaustive consumption of the regenerated mediator *P*. Correspondingly, the first wave gives rise to greater currents than the second.

Second, considering the droplet voltammograms, as the contact angle increases, the peak currents of the first wave increase and shift to more negative potentials. In contrast, the second wave essentially retains its position, albeit with increased current, reflecting the size of the droplet deposit. Recognising that, particularly for the largest contact angle considered, the simulation of the second wave is not completely converged, it is apparent that the second waves for the smaller contact angles considered (*θ* = ¼π and ½π) are effectively modelled by very thin layer behaviour: the magenta and cyan lines almost follow the red and blue lines, respectively. This is in line with our earlier work [53], in which *s* = 1 corresponds to thin layer behaviour. However, strikingly, the depletion regime within the droplets is *non-uniform* both across the electrode surface and droplet volume: the concentration profiles of the reduced mediator, species *P*, taken at *ε* = 10, depicted in Fig. 2c demonstrate the rôle played by the convergent/divergent diffusion regime at the triple phase boundary. For the largest droplet considered (*θ* = ¾π), there is a greater opportunity for diffusive mass transport to occur; convergent diffusion in this regime to the three-phase boundary still takes place, but its effect is weaker, as evident by the linearization of the depletion zone close to the electrode surface in Fig. 2c(iii). This results in the second wave of the yellow trace almost overlapping with that corresponding to planar diffusion in Fig. 2a.

The characteristics of the first peak follow a mix of behaviour corresponding to thin film and planar diffusion. For example, as with the thin film model, the peak current increases with droplet size, with the fast, homogeneous kinetics causing a more facile oxidative electrode reaction. But the potential at which this takes place is more positive compared with the location, ${\varepsilon }_{p}=-\text{In}\left(\lambda \right)$, predicted by the thin film model, and the oxidation becomes increasingly more difficult as the droplet becomes larger (corresponding to increased contact angle). Yet, for the smallest droplet considered (*θ* = ¼π), the peak potential of the first wave approaches that anticipated for planar diffusion in the KT2 region, *viz* ${\varepsilon }_{P}\to 0.409-\frac{1}{2}\text{In}\left(\frac{\lambda }{\gamma }\right)$. The peak currents for the three cases considered exhibit a quadratic relationship (*R*^2^ = 1) with contact angle: ${\psi }_{p}=0.153{\theta }^{2}-0.217\theta +0.160$. Thus, the peak current matches with that expected for planar diffusion, viz.${\psi }_{p}=0.305s\gamma$, only when *θ* = 1.91 (104°). Thus, the first wave cannot be modelled as either one-dimensional diffusion or as a thin film—a result that derives from the convergent/divergent diffusion regime at the triple phase boundary.

**Fig. 3:**
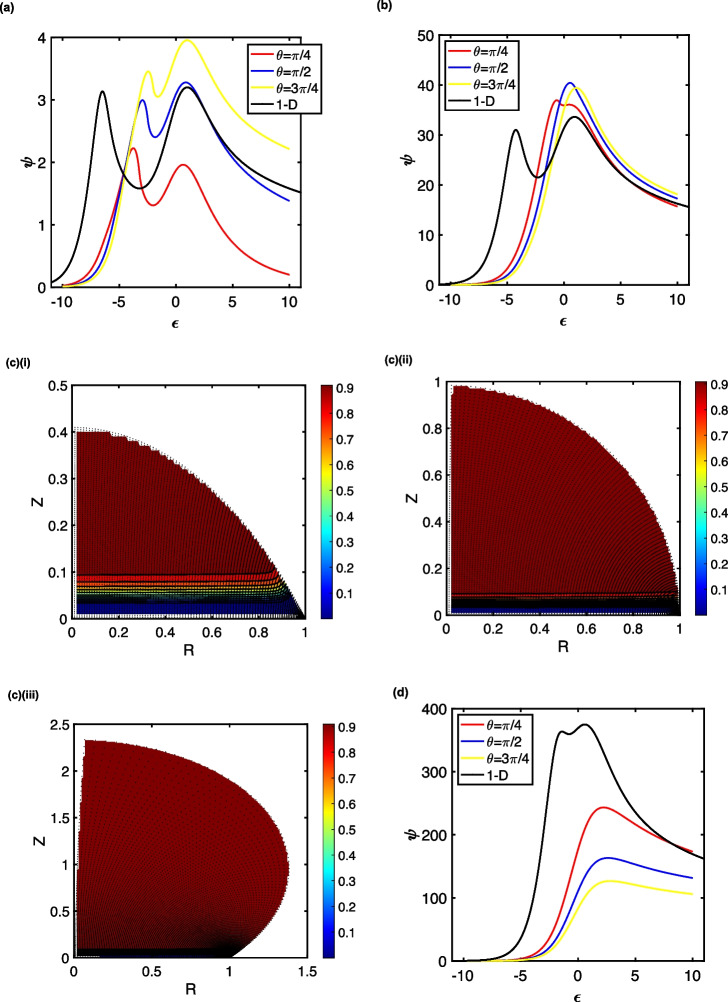
The KT2 zone of the EC’ mechanism in droplets for s = 10, γ = 1, λ = 1.25 x 10^6^, (**a**), which can transition to the KG* zone, depending on the contact angle for s = 100, γ = 1, λ = 1.25 x 10^4^, (**b**). Panel (**c**) illustrates the concentration profiles for the depletion of the substrate (M) at the end of the LSV (ε = 10) for droplets of θ = ¼π, (i); ½π, (ii); and ¾π, (iii). The minimal depletion and diffusive nature of the KG* zone is evident by the re-ordering of the current sizes with contact angle when s = 103, γ = 1, λ = 125, (**d**)

As the diffusion depletion layer becomes smaller than the size of the droplet (*s* > 1), the voltammetric peaks shift towards more positive potentials and become larger, as illustrated in Fig. 3 for *s* = 10 and *s* = 100. Although the planar diffusion case suggests that these conditions correspond to the KT2 zone, the contact angle controls the relative size of the depletion zone *vis-à-vis* that of the droplet, and this can cause the catalytic and redox peaks to overlap, as seen in Fig. 3b for *s* = 100 and *γ* = 1. The concentration profiles in Fig. 3c corresponding to these conditions demonstrate that the substrate (*M*) is consumed by a small amount of the mediator in regions that are only close to the electrode surface. The relative size of the depletion zone to the droplet height controls the transition of the LSV waveshape from the KT2 to the KG* regions: the larger this diffusion zone is compared with the droplet height, the more linear the substrate depletion region is, enabling the contact angle to cause a transition from the KT2 to the KG* region. Yet, despite the linear concentration profiles, the voltammetric characteristics do not correspond to those for planar diffusion. Moreover, it is noteworthy that although these conditions correspond to the KG* region in the classical kinetic zone diagram for the EC’ reaction [87], a split-wave is observed in the planar diffusion simulation, indicative of the KT2 region.

This kinetic zone shift is also marked by the voltammograms changing over from an increase in peak current with contact angle, to one where the peak current *decreases* as the droplet size (contact angle) is increased. This effect is a manifestation of the significance of convergent diffusion (transport) to the three-phase boundary over exhaustive depletion of the substrate (*M*). This is particularly seen in Fig. 3d, when *s* = 1000 and *γ* = 1—conditions that are in the general kinetics (KG*) zone.

Thus, when the excess factor is unity, the total catalysis KT2 regime is still marked by two waves, which cannot be singly considered through the thin wave or planar diffusion models. The droplet geometry (space) and experimental timescale dictate the relative degree to which exhaustion and diffusion interplay, which can lead to a zone-crossover as the contact angle changes.

### The pure kinetics (K), no substrate consumption (KS) and kinetic diffusion (KD) zones

We next turn attention to the case when the excess factor is larger (*γ* = 10^3^), for fast electrode kinetics, with a bimolecular rate constant for the redox catalytic reaction kept at 1.0 × 10^3^ M^−1^ s^−1^ for the simulations (fully converged) with droplets of basal radius and contact angle as in the previous zones.

**Fig. 4:**
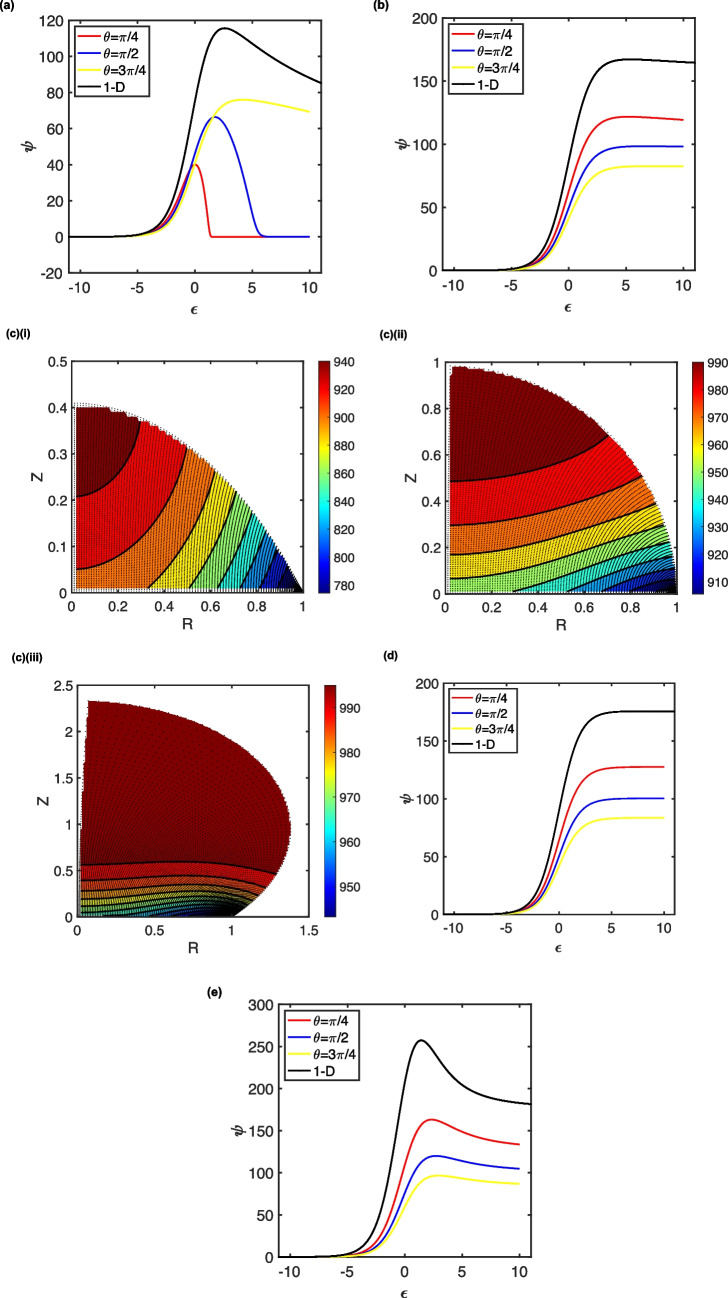
Droplet LSVs corresponding to the K (panels **a** and **b**), KS (panel **d**) and KD (panel **e**) kinetic zones. In (**a**), s = 1, γ = 10^3^, λ = 125; in (**b**), s = 10, γ = 10^3^, λ = 1.25; in (**d**), s = 100, γ = 10^3^, λ = 1.25 x 10^-2^; in (**e**), s = 10^3^, γ = 10^3^, λ = 1.25 x 10^-4^. Panel (**c**) illustrates the concentration profiles for the depletion of the substrate (M) at the end of the LSVs (ε = 10) in (**b**) for droplets of θ = ¼π, (i); ½π, (ii); and ¾π, (iii)

Under these conditions, when *s* = 1, the peak currents are larger than the corresponding case when *γ* = 1, and the peak potentials are shifted more positively. Nevertheless, the peak currents increase with contact angle (droplet size). However, as before, the contact angle can cause the voltammetric behaviour to shift from thin film characteristics to those that illustrate a diffusive characteristic (see Fig. 4a). Nevertheless, the currents are smaller than those given by merely planar diffusion. These voltammograms occupy the pure kinetics (K) zone [87], where there is a degree of substrate depletion close to the electrode surface.

When the parameter *s* is increased to 10 and 100 (Fig. 4b and d, respectively), the voltammetry shifts from the K zone to the KS zone, as only a minimal amount of substrate is depleted close to the electrode. This gives rise to the quasi-steady-state (Fig. 4b) and sigmoidal (Fig. 4d) voltammograms illustrated. As expected for the case where the transport regime is important, the currents decrease with increasing contact angle (drop volume). However, depletion close to the three-phase boundary is highly significant, with the extent of this being larger as the contact angle becomes smaller—see the concentration profiles shown in Fig. 4c.

In KS zone (Fig. 4d), the observed limiting current is a fraction of that expected for planar diffusion, *viz*.${\psi }_{lim}=f\sqrt{\lambda \gamma }$, where 0 < *f* < 1 and fits to $f=0.0480{\theta }^{2}-0.310\theta +0.940$, with *R*^2^ = 1 for *s* = 100, *γ* = 10^3^ and *λ* = 1.25 × 10^−2^. Thus, as $\theta \to 0,{\psi }_{lim}\to 0.940\approx 1$, enabling the one-dimensional diffusion result to be reproduced. It is thus apparent that, if transport within the droplet were to be mistakenly analysed as due to planar diffusion, the apparent rate constant would be a factor of 1/*f* larger than its true value, all other things being equal. Hence, a system with a true rate constant of 1.0 × 10^3^ M^−1^ s^−1^ would appear, in a droplet of *θ* = 2.27 (130°), to be mistakenly reported as 2.1 × 10^3^ M^−1^ s^−1^. This interpreted larger rate constant would afford an inference of an accelerated reaction rate in the droplet compared with that analysed using macroelectrode voltammetry in a semi-infinite solution. Likewise, if it is incorrectly assumed that the dimensionless limiting current for the droplet, *ψ*_lim_, is constant as the droplet size (*a*) changes at constant contact angle, the analysis would suggest ${k}_{EC},\propto \frac{1}{{a}^{2}}$, giving rise to the interpretation that as the droplet size decreases, the homogeneous rate constant increases, in spite of the smaller droplet causing a change in the relative contribution of transient *versus* exhaustive electrolysis: the Fourier number increases as drop size decreases.

When the dimensionless scan rate increases further so that *s* = 1000, the voltammograms are no longer sigmoidal (Fig. 4e). The kinetic parameter, *λ* = 1.25 × 10^−4^, is so small that the conditions move from the KS zone to the kinetic diffusion (KD) zone, where the substrate concentration is not depleted significantly. Although this is again off-the-scale of the conventional kinetic zone diagram [87], the observed peak currents are, nevertheless, smaller than the planar diffusion case and decrease with contact angle (droplet size), indicating the occurrence of a cylindrical diffusion regime caused by the three-phase boundary.

Thus, in the case where the excess factor is very much larger than unity (the substrate concentration is larger than that of the mediator), the classical conditions for electroanalysis of the substrate, the two-dimensional transport regimes are all important, except at the longest of experimental timescales. Yet, still, there is significant non-uniform depletion of the substrate and redox mediator across the electrode and over the droplet volume.

We next turn to the comparison of the EC’ droplet model with the experimental data reported recently [65] for the oxidation of l-cysteine by ferricyanide at pH 6.3–6.5.

### Redox catalytic oxidation of l-cysteine by ferricyanide inside microlitre droplets: comparison of experimental data with the EC’ droplet model

For approximately 25 years, we have been interested in the redox catalytic oxidation of H_2_S and sulphydryl thiols by a variety of electro-generated mediators—all with slightly different formal potentials [73] and stability, in aqueous solution, biphasic systems and in biological media [74–85]. In all cases, the oxidation proceeds by a heterogeneous electron transfer at the electrode surface, such as the oxidation of ferrocyanide to ferricyanide, followed by a rate-limiting homogeneous electron transfer reaction. Both the protonated and deprotonated forms of H_2_S or the sulphydryl thiol compete to be oxidised by the oxidised form of the mediator. S6 outlines the mechanism, which gives rise to the rate law:

3 $$\frac{d{c}_{P}}{dt}={k}_{eff}{c}_{Q}{c}_{RSH,0}$$

where *c*_RSH,0_ is the concentration of l-cysteine in the as-prepared solution, which comprises both protonated and deprotonated thiol moiety, the ratio of which depends on the solution pH. The effective bimolecular rate constant is therefore pH-dependent, ${k}_{eff}=\frac{{k}_{protonated}{c}_{{H}^{+}}+{k}_{deprotonated}{K}_{{a}_{3}}}{{K}_{{a}_{3}}+{c}_{{H}^{+}}}$, and is derived in S6. It contains the bimolecular rate constants for the protonated and deprotonated sulphydryl thiol, as well as the acidity constant for the equilibrium deprotonation of the thiol moiety: pK_a3_ ~ 10.5 at 293 K [88].

Based on voltammetric and steady-state (channel flow) data obtained in 0.1 M borate buffer solutions with 0.5 M KCl at pH > 8, we have estimated [74] that the rate constant for the oxidation of the protonated thiol to be *k*_protonated_ = 2.3 ± 1.0 × 10^3^ M^−1^ s^−1^, whilst that for the deprotonated thiol is *k*_deprotonated_ = 2.5 ± 1.0 × 10^4^ M^−1^ s^−1^ at a temperature of 293 ± 2 K. As expected, the protonated form is oxidised more slowly than the deprotonated species. Similar results were reported for the oxidation of hydrogen sulphide and bisulphide in aqueous media by the hydroxymethylferricenium ion over the range 6.0 ≤ pH ≤ 9.0, with *k*_protonated_ = 375 ± 42 M^−1^ s^−1^ and *k*_deprotonated_ = 2230 ± 116 M^−1^ s^−1^ [85]; other studies using tuned redox potential exhibited similar results [80, 81], and the rate constants for ferricenium-based mediators with H_2_S/HS^−^ appear to be in agreement with observations for ferricyanide redox catalysis with H_2_S/HS^−^ [85].

A recent work [65] studied the redox catalytic oxidation of l-cysteine (RSH) by electrochemically generated ferricyanide inside individual aqueous droplets of volume ~ 1.0 μL and buffered at pH 6.3–6.5, supported with 0.5 M KCl. The substrate, l-cysteine, was in excess: *γ* = 10. The single droplets were immobilised on a glassy carbon electrode and bathed by an immiscible polar organic electrolyte, 0.5 M ^n^Bu_4_NClO_4_ in 1,2-dichloroethane, DCE [65]. At the pH considered, the sulphydryl moiety in the l-cysteine side chain is protonated, and accordingly, it was reported that the rate constant for the reaction is low (490 ± 128 M^−1^ s^−1^). This was shown to be significantly different to that inferred from voltammetry in aqueous solutions not confined to droplets (309 ± 89 M^−1^ s^−1^) [65, 66]. To rationalise the apparent reaction rate acceleration inside the droplet, the authors suggested that some of the l-cysteine, which is above its isoelectric point, adsorbs, at high coverage, at the fluid/fluid interface, and its heterogeneous redox catalytic oxidation at that site takes place [65, 66].

**Fig. 5:**
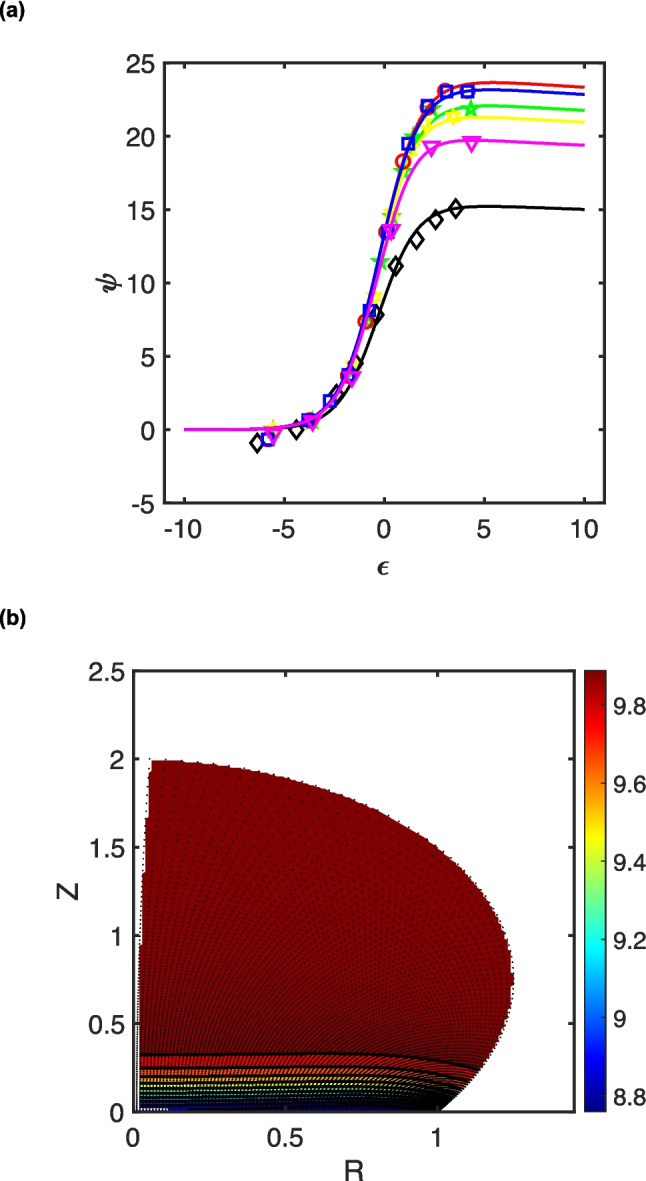
(**a**) LSVs (scan rate of 0.1 V s-1) corresponding to the oxidation of an aqueous ferrocyanide/L-cysteine droplet immobilised on a glassy carbon electrode when immersed into a solution of 0.5 M tetrabutylammonium perchlorate in 1,2 dichloroethane. The composition of the aqueous droplet was 0.5 mM potassium ferrocyanide, 5.0 mM L-cysteine, 50 mM phosphate buffer solution at pH 6.3, and 0.5 mM potassium chloride. Experimental data, manually extracted from voltammograms published in reference [65] and its supplementary information, are illustrated as points. These data are adapted with permission from reference [65]; copyright 2014 American Chemical Society. Key: red circles: Fig. S2g; blue squares: Fig. S2i; green five-pointed stars: Fig 3; yellow six-pointed stars: Fig. S2c; magenta triangles: Fig. S2k; black diamonds: Fig. S2e. Simulated LSVs that best-fit the experimental data are indicated as solid lines (see text and Table 1 for parameters). (**b**) Concentration profile corresponding to the end of the oxidation, illustrating the depletion in the normalised concentration of L-cysteine for the LSV in (**a**) in green

For solutions at pH 6.3, based on the literature data [74], it is expected that the second-order rate constant for the reaction between ferricyanide and l-cysteine is 2.3 ± 1.0 × 10^3^ M^−1^ s^−1^. This value is larger than that estimated in reference [65]. Nevertheless, we chose to extract a rate constant from the droplet voltammograms presented in reference [65], see Fig. 5.

Using the individual droplet photographs in reference [65], together with the dimensions of the basal contacting diameter (2*a*) and the widest part of the droplet (2*r*_d_), the droplet dimensions were estimated assuming a spherical cap model to be *a* ~ 0.5 mm and *h* ~ 1.0 mm (see Table 1), using the trigonometric equations relating the radius of the circular base contact (*a*), with the height of the spherical cap (*h*) and the radius of the sphere (*r*_d_) reported elsewhere [53]. These enabled the contact angle for the aqueous droplet on the glassy carbon electrode and bathed by the organic solvent to be estimated as *θ* ~ 2.28 (~ 130°) and a droplet volume of ~ 1.0 μL, in agreement with that of the aqueous solution carefully pipetted onto the electrode surface in reference [65]. Further, the contact angle calculated using the inferences made above compares favourably with that reported for 1,2-dichloroethane droplets on a glass surface surrounded by water (149 ± 4°) [89]. Moreover, the Eötvös number, $Eo=\frac{\Delta \rho {gr}_{d}^{2}}{{\gamma }_{LL}}$, where the difference between aqueous and organic solvent densities, Δ*ρ* = 0.33 g mL^−1^ [89], the gravitational acceleration is 9.8 m s^−2^, and the tension at the liquid/liquid interface, *γ*_LL_ = 28 mN m^−2^ [89], is calculated to be less than unity. This indicates that, despite the aqueous phase being less dense than the surrounding organic solvent, buoyancy effects in the “skyward” facing droplets do not distort the shape and testify to the veracity of the spherical cap assumption.

**Table 1:** Analysis of experimental data from reference [65]; see Fig. 5

Figure reference [65]	*a*/mm	*h*/mm	*θ*	*V*_droplet_/μL	*E*^0′^/V *vs.* Ag/AgCl	10^−3^ *k*_EC′_/M^−1^ s^−1^
Fig. 3	0.49	1.02	2.63	0.94	0.19	1.1
Fig. S2c	0.55	1.02	2.58	1.03	0.19	0.8
Fig. S2e	0.34	1.09	2.82	0.87	0.21	1.0
Fig. S2g	0.48	1.07	2.67	1.02	0.20	1.4
Fig. S2i	0.50	1.02	2.63	0.95	0.20	1.2
Fig. S2k	0.53	1.07	2.62	1.11	0.19	0.7
*Arithmetic mean*	*0.48*	*1.05*	*2.66*	*0.99*	*0.20*	*1.0*
*Standard deviation*	*0.07*	*0.03*	*0.09*	*0.08*	*0.01*	*0.3*

Voltammetric data across the whole waveshape, extracted manually from the figures reported in reference [65], were normalised by the droplet size (*a*) and composition (*c*^0^_P_ = 0.5 mM; *c*^0^_M_ = 5.0 mM), assuming a temperature of 293 K and ferrocyanide diffusion coefficient of *D*_P_ = 4.6 × 10^−6^ cm^2^ s^−1^ [90], which is in agreement with that employed in our previous work (4.9 × 10^−6^ cm^2^ s^−1^) [74]. Dimensionless potentials were informed by reference [65] as *E*^0′^ ~ 0.20 V *vs.* Ag/AgCl, see Table 1. Given that ferrocyanide voltammetry on glassy carbon exhibits quasi-reversible electrode kinetics [91], we employed the average of the range quoted in reference [91] for the value of the standard heterogeneous rate constant, *k*_s_ = 0.035 cm s^−1^, maintaining the symmetry factor *α* = ½. In this way, single voltammograms for scan rates of 100 mV s^−1^ could be readily computed (Δ*ε* = 10^−4^), with the diffusion coefficients for ferricyanide, *D*_Q_ = 5.1 × 10^−6^ cm^2^ s^−1^ [90] and l-cysteine, *D*_M_ = 2.5 × 10^−5^ cm^2^ s^−1^ [74] taken from previous work. The value of the bimolecular rate constant, *k*_EC′_, was iterated until a good fit was determined, by eye, *q.v.* Figure 5a. In keeping with the notion that buoyancy within the system can be neglected, together with the small experimental timescale $\frac{RT}{Fv}<0.3s$, natural convection inside the droplets resulting from the differential densification coefficients of ferri/ferrocyanide was neglected [92].

The fitted values of the rate constant in the droplets are detailed in Table 1, and average at *k*_EC′_ = 1.0 ± 0.3 × 10^3^ M^−1^ s^−1^, which is in agreement with that expected from semi-infinite voltammetric measurements: there is no apparent significant difference in the bimolecular rate constant for homogeneous electron transfer between ferricyanide and l-cysteine in microlitre droplet and semi-infinite solution. This means that no reaction rate acceleration takes place as a result from this confined chemistry. Whilst this result might have been anticipated given the large volumetric size of the droplet, it is, however, a direct contrast with the interpretation of the data reported in reference [65].

The concentration profile for the depletion of l-cysteine (see Fig. 5b) indicates the importance of cylindrical diffusion driven by the singularity at the triple phase boundary, yet it does not require the adsorption of l-cysteine at the liquid/liquid interface, nor does it necessitate an additional heterogeneous electron transfer thereat. Nevertheless, it is insightful to enquire as to why the experimental data from reference [65] affords an analysis here that is different from that reported in reference [65].

First, whilst it is plausible that l-cysteine above its isoelectric point may behave as a surfactant, there is limited evidence that supports its adsorption at the liquid/liquid interface. The reactive transformation of adsorbed l-cysteine might be expected to give rise to a surface tension gradient at the liquid/liquid interface, which would incur Marangoni transport [53]—effects that have not been reported to have been observed. This might have been in part due to the low concentration of l-cysteine employed (5.0 mM), or possible replacement by the insoluble product (l-cystine) accumulating at the liquid/liquid interface.

Second, we have considered the droplets to be of a spherical cap geometry, and the calculated droplet volume (~ 1.0 μL) matches that reported to have been placed on the electrode surface during experimentation (1.0 μL). In contrast, in reference [65], the assumption is of an oblate ellipsoid cap that has a contact radius of 0.49 mm, widest dimension of 0.63 mm, height above the electrode surface of 0.73 mm and height above the widest point of 0.45 mm. Thus, treating the missing ellipsoidal cap of semi-axes *b* = 0.63 mm and *c* = 0.45 mm, height, *h* = 2*c* − 0.73 = 0.17 mm, the volume of droplet considered [93], ${V}_{drop}={V}_{ellipsoid}-{V}_{cap}=\frac{4}{3}\pi {b}^{2}c-\pi {b}^{2}\left\{\frac{2}{3}c-\left(c-h\right)+\frac{{\left(c-h\right)}^{3}}{3{c}^{2}}\right\}$, is 32% smaller (~ 0.68 μL) than that reported to have been used.

Third, in reference [65], *D*_p_ = *D*_Q_ = 6.5 × 10^−6^ cm^2^ s^−1^, which is slightly smaller than the geometric mean of the diffusion coefficients for ferri/ferrocyanide at 298 K [90], with *D*_M_ = 1.0 × 10^−5^ cm^2^ s^−1^. However, as indicated earlier, all three diffusion coefficients are different in this work. Moreover, we fix the electrode kinetics for the mediator redox couple as on the quasi-reversible/reversible cusp (0.035 cm s^−1^) irrespective of whether the reaction takes place in a confined environment (the mass transfer coefficient inside the droplet is estimated as being *ca.* 0.05 cm s^−1^). However, in reference [65], the electrode kinetics are reported as being (comfortably) reversible in both semi-infinite solution (0.1 cm s^−1^) and when restricted inside an aqueous droplet (1.0 cm s^−1^), albeit with apparent faster electrode kinetics when the electrode surface is exposed to both the aqueous droplet and the 1,2-dichloroethane electrolyte, despite the ferro/ferricyanide redox couple being known to be sensitive to pre-exposure of the electrode to organic solvents [94].

Last, it is curious that the concentration profile in Fig. 5b exhibits similarity with that reported in Fig. 4 of reference [65]. In our system, the homogeneous reaction takes place only inside the droplet and affords a substrate depletion geometry that is similar to that reported in reference [65] where mediated oxidation of both homogenous and heterogeneous forms of the substrate occurs. Our calculations have relied on the modelling of the triple phase boundary using a two-dimensional conformal map of the diffusion space [53, 61] using finite difference methods (*q.v.* S2 and S3), whilst reference [65] employs the adaptive triangulation mesh in a commercial finite element software package. Although the form of the Laplacian in reference [65] is not explicitly identified as being two-dimensional cylindrical space, when that software package employs finite element calculations using a conformal map for the diffusion of species inside a droplet [89], the simulations match up with those we have previously reported [53]. This suggests that, the earlier points notwithstanding, the simulations made in reference [65] were scuppered by the difficulties in the triangulation mesh in accurately describing the flux at the triple phase boundary. As we elaborated in the “Background” section, the accurate modelling of the dynamics at that singularity is notoriously difficult to achieve; adsorption at the liquid/liquid interface is then viewed as an artificial recourse to overcome that issue.

## Conclusions

In this paper, we have characterised homogeneous redox catalysis within electrochemically supported microdroplets immobilised on an electrode surface and bathed by an immiscible electrolyte solution. Linear sweep voltammograms corresponding to five kinetic zones have been illustrated, even under conditions when acute reaction fronts develop. Whilst droplet exhaustion takes place, the voltammetry cannot be described by simple thin layer or semi-infinite diffusion systems, since both the restricted geometry, coupled with the reactivity at the triple phase junction, cause subtle nuances that are compounded by the droplet volume and contact angle: droplet systems afford both spatial and temporal non-uniform depletion zones.

In contrast with literature reports, we have demonstrated that, if convergent mass transport at the three-phase boundary is taken into consideration, the redox catalytic rate constant inside the droplet matches that observed at planar electrodes: *there is no droplet-induced acceleration of the redox catalytic reaction*. Whilst a number of reasons have been provided to explain this discrepancy, we recognise that there is a potential problem in using triangulation meshes in finite element simulations—these do not appear to map out accurately the reactivity at the three-phase junction. This is a profound result, which may have significant quantitative methodological implications for inferences based on numerical simulations wherever such constrained singularities, or menisci, exist, such as in polymer electrolyte fuel cells and electrolysers, molten salt electrometallurgy, electroanalytical pin-prick blood tests, chemical exchange within both geological fluid inclusions and during biological intercellular fusion events, as well as in imaging techniques such as scanning electrochemical cell microscopy (SECCM) [95], and in other, non-electrochemical methods, where reaction rate acceleration in droplets on surfaces has been reported.

## Supplementary Information

Below is the link to the electronic supplementary material.

Supplementary file1 EC’ reaction under semi-infinite, droplet and thin layer conditions, computational methods including algorithms used (PDF 2199 KB)

Supplementary file2 (PDF 187 KB)

Supplementary file3 (PDF 189 KB)
