# Supplementary material for: Are redox catalytic reaction rates accelerated in microdroplets on electrode surfaces?
Source: J Solid State Electrochem. 2025 Apr 12;29(6):2321–34. doi: 10.1007/s10008-025-06283-4 (PMC12103339; doi:10.1007/s10008-025-06283-4)
Supplement: Supplementary file 1 — Supplementary file1 EC’ reaction under semi-infinite, droplet and thin layer conditions, computational methods including algorithms used (PDF 2199 KB) [file 10008_2025_6283_MOESM1_ESM.pdf]

Are Redox Catalytic Reaction Rates Accelerated in Microdroplets on Electrode Surfaces?

Nathan S. Lawrence,* Jay D. Wadhawan*

School of Engineering, Chemical Engineering,
The University of Hull, Cottingham Road,
Kingston-upon-Hull HU6 7RX, United Kingdom.

*Corresponding authors
E.mail: nathan.lawrence@hull.ac.uk (NSL); j.wadhawan@hull.ac.uk (JDW)

Supporting Information

S1: *Overview of the EC' Reaction* S2
S2: *EC' Reaction of Droplets on Electrodes* S6
S3: *Computational Strategy and Methods* S11
S4: *A Heptadiagonal Matrix Algorithm* S13
S5: *EC' Reaction in a Very Thin Layer* S15
S6: *The Mediated Oxidation of H_2S or Sulphydryl Thiols* S17
S7: *References* S18

S1. Overview of the EC' Reaction

The classical scheme for electrochemically mediated redox catalysis in homogeneous solution (EC' reaction) involves both heterogeneous electron transfer through activation of the mediator, P, to form reactive species Q, and the subsequent homogeneous electron transfer between the latter species and the substrate, as given in the typical one-electron, stepwise mechanism, in equation (1) [S1-S21]:

$$\begin{aligned} &P - e^- \underset{k_b}{\overset{k_f}{\rightleftarrows}} Q \\ &Q + M \xrightarrow{k_{EC'}} P + N \qquad \text{(S1.1)} \\ &N \xrightarrow{fast} \text{Products} \end{aligned}$$

We describe the reduced form of the mediator as P, the oxidised form of the mediator as Q, and the substrate (or analyte) as M. Note that our description here differs slightly from that employed conventionally, in that we denote the substrate as M, as opposed to A.

In this mechanism, the heterogeneous electron transfer reaction can be considered to follow Butler-Volmer kinetics, with a standard heterogeneous rate constant of k_s and a transfer coefficient of α, and with a formal electrode potential of $E^{0'}$. The homogeneous electron transfer step is considered to be rate-determining (rate constant $k_{EC'}$) and irreversible, so that the follow-on reaction is fast, and can be neglected.

As noted by Feldberg and Campbell [S16], this positive feedback loop mechanism, is quite rare in chemistry, as it demands that (1) the substrate should be easier to oxidise than the mediator, so as to ensure that the homogeneous step is thermodynamically "downhill"; (2) the heterogeneous kinetics associated with the direct oxidation of the substrate must be slower than those of the mediator, so as to ensure that this "side reaction" does not take place to any significant extent; and (3) the homogeneous reaction kinetics are sufficiently rapid, to ensure that the catalytic effect is significant under the voltammetric timescales employed. Feldberg and Campbell found that the fulfilment of all of these conditions is easier when the diffusion coefficient of the substrate is much smaller than that for that of the mediator

[S16], echoing earlier theoretical and experimental work by Hapiot *et al.*, who also explored the voltammetry when the diffusion coefficients of the mediator and the substrate are different [S5,S6]. Murray also explored this catalytic reaction in polymeric solution, where it is easier to observe differences in diffusion coefficients [S3,S13].

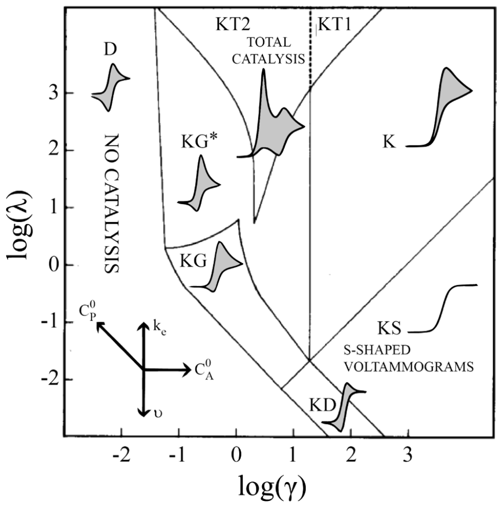

Fig. S1.1: *Kinetic zone diagram for the EC' reaction at planar electrodes developed by Savéant and Su [S4], depicted here from work by Dempsey* et al. *[S17]. Briggsian logarithms are employed, with the catalytic rate constant denoted as k_e, with the mediator and substrate concentrations in bulk solution described by C_P^0 and C_A^0, respectively. All other symbols are commensurate with those described in this work. Figure reprinted with permission from reference [S17]; copyright 2014 American Chemical Society.*

For the classical case of all species having equal diffusion coefficients (D_P) and fast electrode kinetics, Savéant and Su [S4] outlined the kinetic zone diagram (see Figure S1.1) that encompasses all forms of EC' behaviour for cyclic voltammetry at planar electrodes of area S (*viz.* where one-dimension diffusional transport of chemical species to and from the heterogeneous interface takes place). Two very special limiting cases are observable, which are of importance for electrolytic manufacturing (or redox catalysed fuel cells) and sensing. For the latter, the KS zone is associated with sigmoidal-shaped voltammograms, $\Psi = \dfrac{i}{FSc_P^0\sqrt{D_P}\sqrt{\dfrac{Fv}{RT}}} = \dfrac{\sqrt{\dfrac{RT}{Fv}}\sqrt{k_{EC'}c_M^0}}{1+\exp\left\{\dfrac{F}{RT}\left(E-E^{0'}\right)\right\}}$, where the symbols are defined in Table S1.1. These voltammograms exhibit a current plateau that is directly proportional to the square root of the bulk concentration of the

Table S1.1: *Descriptions of the general variables for EC' reactions employed in this work*

Symbol	Description
c^0_i	Concentration of species i = P, Q or M in bulk solution, or initial concentration of species i.
D_i	Diffusion coefficient for species i = P, Q or M
E	Applied potential
E^0	Formal potential of the P/Q redox couple
E_p	Peak potential
$E_{½}$	Half-wave potential
F	The Faraday constant (96485.3 C mol^{-1})
i	Current flowing between working and counter electrodes in a potentiostatic circuit. This is assumed to be the Faradaic current only.
$k_{EC'}$	Homogeneous rate constant for the redox catalysis reaction.
k_s	Heterogeneous rate constant (Butler-Volmer kinetics)
R	Molar gas constant (8.3145 J mol^{-1} K^{-1})
S	Geometric area of the electrode
T	Absolute temperature
t	Time
u	Dimensionless potential at the start of a voltammetric sweep
v	Voltammetric scan rate
α	Symmetry factor (Butler-Volmer kinetics)
β	Concentration corresponding to one standard addition of the analyte
Λ_{MA}	Matsuda-Aybe parameter
Ψ	Dimensionless current for the case of planar diffusion transport.

substrate, and with a half-wave potential centred on the formal potential for the mediator redox couple ($E_{½} = E^{0'}$). Under this regime, because the concentration ratio of the substrate (c_M^0, analyte) to that of the mediator (c_P^0) in bulk solution is large, $\lg\gamma = \lg\left(\frac{c_M^0}{c_P^0}\right) \geq 2$, and the kinetic factor (Damköhler number) is relatively small, $\lg\lambda = \lg\left(\frac{RT}{Fv}k_{EC'}c_P^0\right) \leq 0$, the concentration of the substrate is essentially constant throughout the homogeneous solution, and the system is appears to be in steady-state: no substrate is effectively consumed [S4]: $\frac{\lambda}{\gamma} \to 0$, and the limiting current is given by $\Psi_{\lim} = \sqrt{\frac{RT}{Fv}}\sqrt{k_{EC'}c_M^0} = \sqrt{\lambda\gamma}$. For analytical detection purposes, this is the ideal case: when small additions (β) of the analyte are made, the square root term can be linearised, *viz.* $\sqrt{c_M^0 + \beta} \approx \sqrt{c_M^0} + \frac{1}{2\sqrt{c_M^0}}\beta$, enabling classical, "standard additions" plots [S22], whilst the S-shaped voltammetric curve necessarily affords the advantage of a large signal-to-noise ratio.

The other important zone is the KT region, corresponding to total catalysis [S4]. This typically occurs when the concentrations of substrate and mediator are comparable, $\lg\gamma = \lg\left(\frac{c_M^0}{c_P^0}\right) \sim 0$, and the kinetic factor is relatively large, $\lg\lambda = \lg\left(\frac{RT}{Fv} k_{EC'} c_P^0\right) \geq 2$. Under these conditions, the consumption of the substrate is large [S4]: $\frac{\lambda}{\gamma} \to \infty$, so that its transport is the main controlling factor. This is the ideal case for mediated electrochemical manufacture, or for maximising the consumption of the feed in a fuel cell. However, it is not without its own nuances: for lower excess factors, $\gamma = \frac{c_M^0}{c_P^0}$, split-wave voltammograms occur (KT2 zone), with the peak current of the first wave being independent of the reaction kinetics, $\Psi_p = 0.609\gamma$, since only a tiny amount of heterogeneous mediator transformation is required for the complete homogeneous consumption of the substrate. However, the peak potential of the first wave shifts to indicate that the heterogeneous electron transfer process becomes more facile with increased catalytic rate constant, $\varepsilon_p = \frac{F}{RT}\left(E_P - E^{0'}\right) = 0.409 - \frac{1}{2}\ln\left(\frac{\lambda}{\gamma}\right)$, with the half-peak width, $\varepsilon_p - \varepsilon_{p/2} = 1.14$. The second wave, however, essentially corresponds to the planar diffusion of the mediator, and appears at the potential where no homogeneous reaction occurs [S4]. On increasing the excess factor to $\gamma \geq 20$, the second wave becomes sufficiently small relative to the first, so that only one wave appears (KT1 zone). Thus, for application in electrosynthesis, or electrochemical remediation, both KT1 and KT2 zones are important, so that a large amount of feedstock, or effluent, is transformed into products.

We next develop the theory corresponding to the EC' reaction mechanism occurring in droplet-modified electrodes under diffusion control, and where ion transfer between the droplet and its bathing fluid does not constrain the Faradaic process occurring at the electrode.

S2. EC' Reactions in Droplets on Electrodes

We consider an EC' reaction, equation (S1.1), that occurs *inside* a single, spherical cap shaped oil droplet (of basal radius a, and height above the surface, h) that is fully electrochemically supported, and immobilised on an electrode surface, such that the contact angle is θ, see Figure S2.1. In this way, as illustrated previously [S23], although heterogeneous electron transfer occurs only through the circular basal disc of the droplet that is in contact with the electrode, it is convenient to split this into two regions comprising the triple phase boundary (the toroidal volume whose thickness is *ca.* 2‰ of the contact radius, located where solid electrode, liquid aqueous electrolyte and liquid oil droplet meet) and the remainder of the contacting disc [S23,S24]. We assume that the Eötvös number of the droplets is smaller than unity so that gravitational effects are negligible *vis-à-vis* surface tension effects; this is true for oil droplets bathed by water, and under typical experimental conditions, *viz.,* a ~ 1 - 1000 μm, D_P ~ 10^{-6} – 10^{-5} $cm^2\ s^{-1}$, v = 100 mV s^{-1} and T = 293 K.

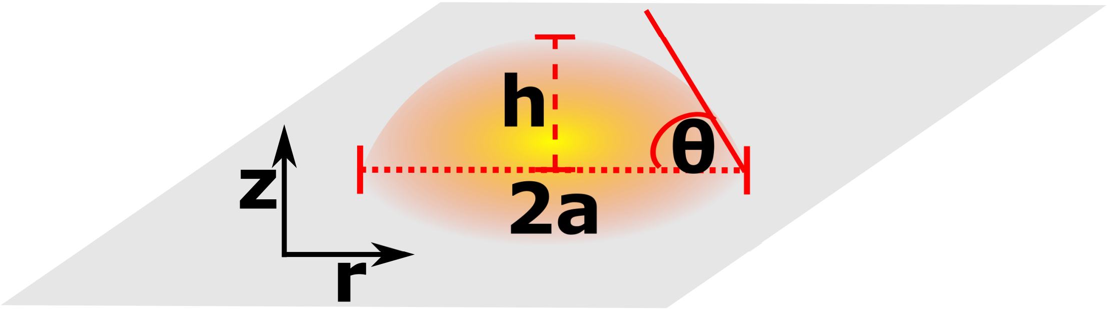

Fig. S2.1: *The geometry of the droplet-modified electrode considered in this work.*

Thus, using the Amatore-Svir-Olenick conformal transformation [S24],

$$R = -\frac{\sinh\left\{\frac{\pi}{2}\tan\left(\frac{\pi}{2}\eta\right)\right\}}{\cosh\left\{\frac{\pi}{2}\tan\left(\frac{\pi}{2}\eta\right)\right\} - \sin\left(\frac{\pi}{2}\xi\right)} \quad \text{(S2.1a)}$$

$$Z = \frac{\cos\left(\frac{\pi}{2}\xi\right)}{\cosh\left\{\frac{\pi}{2}\tan\left(\frac{\pi}{2}\eta\right)\right\} - \sin\left(\frac{\pi}{2}\xi\right)} \quad \text{(S2.1b)}$$

the space occupied by the droplet is readily mapped onto a rectangular grid comprising [ξ,η] orthonormal co-ordinates: $-1 \le \xi \le \frac{2}{\pi}\theta - 1, -0.86 \le \eta \le 0$, where the lower bound for η is sufficiently small to afford a negligible geometric error, whilst maintaining a fast computation [S24].

In equation (S2.1), R is the cylindrical co-ordinate that is tangential to the electrode surface, with Z being that perpendicular to the surface, both being reduced through normalisation by the radius of the basal disc of the droplet (a), whilst recognising the axial symmetry of the system:

$$R = \frac{r}{a} \qquad \text{(S2.2a)}$$

$$Z = \frac{z}{a} \qquad \text{(S2.2b)}$$

In this work, we only consider mass transport through diffusion, and assume that any ion transfer across the liquid/liquid interface that accompanies the Faradaic process at the electrode, to maintain electroneutrality within the droplet, is fast. Thus, the diffusive transport of species P, Q and M within the droplet is given by the expressions in equation (S2.3),

$$\frac{\partial p}{\partial \varepsilon} = \lambda_1 \frac{\partial^2 p}{\partial \xi^2} + \lambda_2 \frac{\partial^2 p}{\partial \eta^2} + \lambda_3 \frac{\partial p}{\partial \eta} + \lambda_4 \frac{\partial p}{\partial \xi} + \lambda qm \qquad \text{(S2.3a)}$$

$$\frac{\partial q}{\partial \varepsilon} = \lambda_{1q} \frac{\partial^2 q}{\partial \xi^2} + \lambda_{2q} \frac{\partial^2 q}{\partial \eta^2} + \lambda_{3q} \frac{\partial q}{\partial \eta} + \lambda_{4q} \frac{\partial q}{\partial \xi} - \lambda qm \qquad \text{(S2.3b)}$$

$$\frac{\partial m}{\partial \varepsilon} = \lambda_{1m} \frac{\partial^2 m}{\partial \xi^2} + \lambda_{2m} \frac{\partial^2 m}{\partial \eta^2} + \lambda_{3m} \frac{\partial m}{\partial \eta} + \lambda_{4m} \frac{\partial m}{\partial \xi} - \lambda qm \qquad \text{(S2.3c)}$$

in which the adimensional variables are defined in Table S2.1.

Note that the expressions in equation (S2.3) involve transport within two dimensions. This is important, since it enables the flux at the three-phase boundary to be estimated.

Table S2.1: *Descriptions of the dimensionless variables employed for the transport equations*

Reduced Variable	Equation	Symbol Definitions
Concentration	$p=\frac{c_P}{c_P^0};\ q=\frac{c_Q}{c_p^0};\ m=\frac{c_M}{c_M^0}$	Concentrations of the three species P, Q and M, each normalised by the initial bulk droplet concentration of species P, c_P^0
Time	$\tau=\frac{D_P t}{a^2}$	Dimensionless time, τ
Potential	$\varepsilon=\frac{F}{RT}\left(E-E^{0'}\right)$	Dimensionless potential, ε
Space-time variable (reduced scan rate)	$s=\frac{a}{\sqrt{D_P}}\sqrt{\frac{Fv}{RT}}$	Fourier number: $\frac{1}{s^2}=\frac{d\tau}{d\varepsilon}$
Excess factor	$\gamma=\frac{c_M^0}{c_P^0}$	Excess factor, γ
Relative diffusivities	$d_Q=\frac{D_Q}{D_P}\ ;\ d_M=\frac{D_M}{D_P}$	Reduced diffusion coefficients, d_Q and d_M
Normalised rate constant	$\lambda=\frac{RT}{Fv}k_{EC'}c_P^0=\frac{a^2}{D_P s^2}k_{EC'}c_P^0$	Dimensionless rate constant (Damköhler number: ratio of voltammetric timescale to reaction timescale)
Normalised current	$\psi=\frac{i}{2\pi F D_P a c_P^0}$	Dimensionless current, $\psi=\frac{1}{2}s\Psi$
Transport coefficients	$\lambda_1=\frac{\Delta}{s^2};\ \lambda_{1q}=d_Q\lambda_1;\ \lambda_{1m}=d_M\lambda_1$<br>$\lambda_2=\alpha_t\lambda_1;\ \lambda_{2q}=\alpha_t\lambda_{1q};\ \lambda_{2m}=\alpha_t\lambda_{1m}$<br>$\lambda_3=-\beta_t\lambda_1-\frac{\Lambda}{s^2}\delta_t;$<br>$\lambda_{3q}=-\beta_t\lambda_{1q}-d_Q\frac{\Lambda}{s^2}\delta_t;$<br>$\lambda_{3m}=-\beta_t\lambda_{1m}-d_M\frac{\Lambda}{s^2}\delta_t$<br>$\lambda_4=\frac{\Lambda}{s^2}\gamma_t;\ \lambda_{4q}=\frac{\Lambda}{s^2}\gamma_t d_Q;$<br>$\lambda_{4m}=\frac{\Lambda}{s^2}\gamma_t d_M$	$\Delta=\frac{4}{\pi^2}\left[\cosh\left\{\frac{\pi}{2}\tan\left(\frac{\pi}{2}\eta\right)\right\}-\sin\left(\frac{\pi}{2}\xi\right)\right]^2$<br>$\Lambda=\sqrt{\Delta}$<br>$\alpha_t=\frac{4}{\pi^2}\cos^4\left(\frac{\pi}{2}\eta\right)$<br>$\beta_t=\frac{2}{\pi}\sin\left(\pi\eta\right)\cos^2\left(\frac{\pi}{2}\eta\right)$<br>$\gamma_t=\cos\left(\frac{\pi}{2}\xi\right)$<br>$\delta_t=\frac{2}{\pi}\gamma_t^2\left[\sin\left(\frac{\pi}{2}\xi\right)\coth\left\{\frac{\pi}{2}\tan\left(\frac{\pi}{2}\eta\right)\right\}-\operatorname{csch}\left\{\frac{\pi}{2}\tan\left(\frac{\pi}{2}\eta\right)\right\}\right]$

The relevant boundary conditions involving Butler-Volmer electrode kinetics for the P/Q redox couple, are outlined in Table S2.2, with the initial potential, E_i, given as $u=\varepsilon_i=\frac{F}{RT}\left(E-E_i\right)$.

Table S2.2: *Boundary conditions for EC' voltammetry within a spherical cap droplet.*

Space/Time Descriptor	Boundary Space/Time Location	Dimensionless Conditions
Initial conditions	$\varepsilon \le u,$<br>$-1 \le \xi \le \frac{2}{\pi}\theta - 1,$<br>$-0.86 \le \eta \le 0$	$p=1$<br>$q=0$<br>$m=\gamma$
Liquid/liquid interface	$\varepsilon > u,$<br>$\xi = \frac{2}{\pi}\theta - 1,$<br>$\forall \eta$	$\left(\frac{\partial j}{\partial \xi}\right)=0 \ with\ j=p\ or\ q\ or\ m$
Axis of reflection (symmetry axis)	$\varepsilon > u$<br>$\forall \xi$<br>$\eta = 0$	$\left(\frac{\partial j}{\partial \eta}\right)=0 \ with\ j=p\ or\ q\ or\ m$
Electrode surface:	$\varepsilon > u$	
Three-phase boundary	$-1 \le \xi \le \frac{2}{\pi}\theta - 1,$<br>$\eta = -0.86$	$\left(\frac{\partial p}{\partial \eta}\right)=-d_Q\left(\frac{\partial q}{\partial \eta}\right)=K_s^{'}\left(pe^{\varepsilon}-q\right),\left(\frac{\partial m}{\partial \eta}\right)=0$<br>$with\ K_s^{'}=\frac{\pi^2}{4}\frac{a}{D_P}\frac{k_s e^{-\alpha\varepsilon}\sec^2\left(-0.43\pi\right)}{\left[\cosh\left\{\frac{\pi}{2}\tan\left(-0.43\pi\right)\right\}-\sin\left(\frac{\pi}{2}\xi\right)\right]}$
Main electrode	$\xi = -1$<br>$\forall \eta$	$\left(\frac{\partial p}{\partial \xi}\right)=-d_Q\left(\frac{\partial q}{\partial \xi}\right)=K_s\left(pe^{\varepsilon}-q\right),\left(\frac{\partial m}{\partial \xi}\right)=0$<br>$with\ K_s=\frac{\pi}{2}\frac{a}{D_P}\frac{k_s e^{-\alpha\varepsilon}}{\left[\cosh\left\{\frac{\pi}{2}\tan\left(\frac{\pi}{2}\eta\right)\right\}+1\right]}$

The current (i) flowing at each applied potential is determined through the summation of the contributions at the main electrode and three-phase boundary [S23],

$$\psi = \frac{i}{2\pi F D_P a c_P^0}$$

$$= -3.4225\times10^{-5}\theta\left\{563.5141-\sin\left(\frac{\pi}{2}\xi\right)\right\}\left(\frac{\partial p}{\partial \eta}\right)_{\eta=-0.86} \tag{S2.4}$$

$$-\frac{\pi}{2}\int_{\eta=-0.86}^{0}\frac{\sec^2\left(\frac{\pi}{2}\eta\right)\sinh\left\{\frac{\pi}{2}\tan\left(\frac{\pi}{2}\eta\right)\right\}}{\cosh\left\{\frac{\pi}{2}\tan\left(\frac{\pi}{2}\eta\right)\right\}+1}\left(\frac{\partial p}{\partial \xi}\right)_{\xi=-1}d\eta$$

This enables cyclic voltammograms to be computed for any contact angle, set of diffusion coefficients for species P, Q and M, and for different electrode kinetics; the Matsuda-Ayabe heterogeneous parameter for semi-infinite voltammetric conditions [S25] is $\Lambda_{MA} = \frac{k_s}{\sqrt{D_Q^{1-\alpha}D_P^{\alpha}\frac{Fv}{RT}}} = \frac{k_s a}{s\sqrt{D_Q^{1-\alpha}D_P^{\alpha+1}}}$. Thus, for k_s = 3.1 cm s^{-1}, α = ½, a droplet of basal radius a = 0.5 mm in which all species have equal diffusion coefficients gives electrochemically reversible voltammograms as the dimensionless scan rate (s^2) varies over $1 \le s \le 1000$ (*vide infra*), *i.e.* $\Lambda_{MA} > 15$. As indicated in the main text, the value of k_s chosen for fast electrode kinetics is also upheld for reactions within droplets. As s increases further, the electrode kinetics become increasingly sluggish, as the quasi-reversible, and subsequently irreversible behaviour is approached.

However, there is a subtle change in the diffusion regime that also takes place: thin film behaviour occurs when the droplet size is smaller than, or comparable with, the extent of the diffusive depletion zone (the Fourier number is, at least, unity). Decreasing the value of the Fourier number causes first planar diffusion, then cylindrical diffusion, and ultimately reaches a regime corresponding to a quasi-steady-state [S23]. The exact change in the diffusion regime is dependent on the drop shape, and therefore the contact angle.

Accordingly, we are interested in the effect of these two parameters (s and θ) on the voltammetric waveshape for the EC' reaction with fast electrode

kinetics where all diffusion coefficients are equal, and therefore consider the case of two bimolecular rate constants: $k_{EC'} = 10^9$ M^{-1} s^{-1} (which is close to the diffusion limit) and $k_{EC'} = 10^3$ M^{-1} s^{-1}, with $\frac{\pi}{4} \leq \theta \leq \frac{3\pi}{4}$ and $10^0 \leq s \leq 10^3$ to enable the exploration of five of the kinetic zones in Figure S1.1: KT2 (high λ, low γ), KS (low λ, high γ), K (high λ, high γ), KG* (high λ, low γ) and KD (low λ, high γ). The resulting voltammgrams are illustrated and analysed in the main text.

S3. Computational Strategy and Methods

For comparison with the kinetic zone diagram illustrated in Figure S1.1, voltammograms were simulated under conditions corresponding to fast electrode kinetics under a one-dimensional, planar diffusion regime (all diffusion coefficients set at 10^{-5} cm^2 s^{-1}) at an electrode of radius $r_0 = a = 0.5$ mm, with $k_{EC'}$ fixed at either 10^3 or 10^9 M^{-1} s^{-1} and $\gamma = 1$ or 10^3, and both c^0_P and the temperature fixed at 0.5 mM and 293 K, respectively, with the parameter s in the range $1 \leq s \leq 1000$. The protocols employed were identical to those described previously [S26]. However, the acute reaction front [S27] that develops when the fastest homogeneous rate constant is examined under the slowest value of the dimensionless scan rate necessitated the deployment of a dense spatio-temporal grid for numerical simulations to converge. This involved the use of 2000 temporal nodes and 5000000 spatial nodes, over the box size of 100 diffusion lengths ($100\sqrt{\pi D \frac{RT}{Fv}}$). Note that for comparison with droplets, the dimensionless current in the droplet (ψ) is related to that at a macroelectrode (Ψ) through $\psi = \frac{1}{2} s \Psi$.

The numerical simulation of linear sweep voltammograms in the droplets, with contact angles set at $\theta = \frac{1}{4}\pi$ $\frac{1}{2}\pi$ and $\frac{3}{4}\pi$, was undertaken *via* program encoding in GNU FORTRAN using the freely available gfortran compiler, with double-precision variables. Compiled programs were executed on a

MacBook Air computer running with a 1.3 GHz Intel Core i5 processor, with 4 GB of DDR3 RAM at 1600 MHz speed. These simulations employed a finite difference spatial grid involving 100 x 100 nodes, using centred differences for spatial terms and forward differences for temporal gradients, and the ADI method was used to solve the transport equations. The terms involving bimolecular kinetics were linearised, as reported in previous work [S26], with the concentrations of all three chemical species being simulated simultaneously, using the heptadiagonal matrix algorithm outlined in S4. Simulations used to compare with the kinetic zone diagram employed the values of the parameters described earlier, with $k_s = 3.1$ cm s^{-1} and the symmetry factor maintained at ½. As described previously, these conditions enable $\Lambda_{MA} > 15$ for $1 \leq s \leq 1000$, *i.e.* reversible voltammograms for all scan rates considered. Currents were calculated over a dimensionless potential range of 20, through trapezoidal quadrature. Note that the recommended practice [S24] of averaging the reduced current over the entire length of the three-phase boundary arc was employed. Data were imported using Matlab R2019b (Mathworks) for graphical processing.

A uniform potential step was used for linear sweep voltammetry, which, for most simulations was maintained at $\Delta\varepsilon = 10^{-4}$, giving rise to computation times of *ca.* 70 min. However, lengthier computations were needed in the presence of acute reaction fronts [S27]. Thus, for $k_{EC'} = 10^3$ M^{-1} s^{-1}, s = 1 and $\gamma = 10^3$, converged simulations (*vide infra*) required $\Delta\varepsilon = 10^{-5}$, and computation times ~12 h. Complete convergence was not found for simulations when $k_{EC'} = 10^9$ M^{-1} s^{-1}, s = 1 and $\gamma = 1$, even for $\Delta\varepsilon = 10^{-6}$ (computing times ~ 5 days). Nevertheless, simulations are reported for at least 90% convergence, to understand qualitative effects. Although adaptive gridding [S27] might have been able to shorten computation times and improve convergence, these were not employed.

The assessment of the degree of convergence of the simulations, particularly for s = 1 was undertaken through the integrated charge. For the one-electron transfers considered in this work, the maximum charge transferred, q_0,

corresponding to complete oxidation of the redox mediator is given by Faraday's laws, $q_0 = c_P^0 V_{drop} F = \frac{1}{3}\pi F a^3 c_P^0 \frac{\sin\theta\left(2+\cos\theta\right)}{\left(1+\cos\theta\right)^2}$. Since the instantaneous charge, q, at any point in an uncatalysed voltammogram is given by integration of the voltammogram, *viz.*, $q = 2\pi F D_p a c_P^0 \frac{RT}{Fv}\int \psi \, d\varepsilon$, the fractional charge, Ω, is given by $\Omega = \frac{q}{q_0} = \frac{6}{s^2}\frac{\left(1+\cos\theta\right)^2}{\sin\theta\left(1+\cos\theta\right)}\int \psi \, d\varepsilon$. When redox catalysis takes place, the maximum charge corresponding to exhaustive oxidation of the redox active material droplet, q', is given as $q' = FV_{drop}\left(c_P^0 + c_M^0\right) = c_P^0 V_{drop} F\left(1+\gamma\right)$. Thus, the fraction of the charge passed per equivalent of the mediator catalyst is given as the function $\frac{\Omega}{1+\gamma}$. For complete exhaustion of the reduced form of the redox mediator and the reacting substrate, $\frac{\Omega}{1+\gamma}$ varies smoothly from 0 to 1, with the dimensionless potential. This enables the assessment of the turnover number [S26], as well as, for exhaustive electrolysis, the extent to which the numerical simulations are converged.

S4. A Heptadiagonal Matrix Algorithm

We are interested in solving the diffusion equations corresponding to the three different species P, Q and M simultaneously. However, these three equations are coupled through the homogeneous kinetics: the equation for species P has a kinetic term involving both species Q and M; that for species Q involves species M, and likewise for species M. The boundary conditions are also coupled through the heterogeneous electrode kinetics, particularly for quasi-reversible redox couples, or when the diffusion coefficients of P and Q differ substantially, as is the case for the ferri/ferrocyanide redox couple. A rapid way to solve these coupled equations is to extend the Thomas algorithm (LU decomposition of a tridiagonal matrix) to accommodate for a seven-diagonal

matrix, where the vector of unknown concentrations interleaves and concatenates the three species (P, Q and M) for each variation in the spatial nodes.

Thus, we consider the solution of N simultaneous equations written in the form,

$$\left[T\right]\left\{u\right\}=\left\{h\right\} \tag{S4.1}$$

where the heptadiagonal matrix [T] is of known coefficients, the vector {h} comprises known values, and {u} is the vector of unknown concentrations. Defining the seven-diagonal matrix [T] as:

$$\left[T\right]=\begin{bmatrix}
a_1 & b_1 & c_1 & d_1 & 0 & . & . & . & . & . & . & . & 0 \\
e_2 & a_2 & b_2 & c_2 & d_2 & 0 & . & . & . & . & . & . & 0 \\
f_3 & e_3 & a_3 & b_3 & c_3 & d_3 & 0 & . & . & . & . & . & 0 \\
g_4 & f_4 & e_4 & a_4 & b_4 & c_4 & d_4 & 0 & . & . & . & . & 0 \\
. & . & . & . & . & . & . & . & . & . & . & . & . \\
. & . & . & . & . & . & . & . & . & . & . & . & . \\
0 & . & 0 & g_i & f_i & e_i & a_i & b_i & c_i & d_i & 0 & . & 0 \\
. & . & . & . & . & . & . & . & . & . & . & . & . \\
. & . & . & . & . & . & . & . & . & . & . & . & . \\
0 & . & . & . & . & 0 & g_{N-3} & f_{N-3} & e_{N-3} & a_{N-3} & b_{N-3} & c_{N-3} & d_{N-3} \\
0 & . & . & . & . & . & 0 & g_{N-2} & f_{N-2} & e_{N-2} & a_{N-2} & b_{N-2} & c_{N-2} \\
0 & . & . & . & . & . & . & 0 & g_{N-1} & f_{N-1} & e_{N-1} & a_{N-1} & b_{N-1} \\
0 & . & . & . & . & . & . & . & 0 & g_N & f_N & e_N & a_N
\end{bmatrix} \tag{S4.2}$$

with vectors {u} and {h} being:

$$\left\{u\right\}=\begin{Bmatrix} u_1 \\ u_2 \\ u_3 \\ u_4 \\ . \\ . \\ u_i \\ . \\ . \\ u_{N-3} \\ u_{N-2} \\ u_{N-1} \\ u_N \end{Bmatrix};\quad \left\{h\right\}=\begin{Bmatrix} h_1 \\ h_2 \\ h_3 \\ h_4 \\ . \\ . \\ h_i \\ . \\ . \\ h_{N-3} \\ h_{N-2} \\ h_{N-1} \\ h_N \end{Bmatrix} \tag{S4.3}$$

we solve for {u}:

$$\left[T\right]=\left[T_L\right]\left[T_U\right] \quad \Rightarrow\left\{u\right\}=\left[T_U\right]^{-1}\left(\left[T_L\right]^{-1}\left\{h\right\}\right) \tag{S4.4}$$

by first defining the following parameters using forward recursion, as appropriate.

$$w_1=a_1;\ x_1=b_1;\ y_1=c_1;\ m_1=h_1$$

$$z_2=\frac{e_2}{w_1};\ w_2=a_2-z_2x_1;\ x_2=b_2-z_2y_1;\ y_2=c_2-z_2d_1;\ m_2=h_2-z_2m_1$$

$$\begin{aligned}&\alpha_3=\frac{f_3}{w_1};\ z_3=\frac{e_3-\alpha_3x_1}{w_2};\ w_3=a_3-z_3x_2-\alpha_3y_1;\\&x_3=b_3-z_3y_2-\alpha_3d_1;\ y_3=c_3-z_3d_2;\ m_3=h_3-z_3m_2-\alpha_3m_1\\&\mathit{for}\ i=4\rightarrow N,\\&\beta_i=\frac{g_i}{w_{i-3}};\ \alpha_i=\frac{f_i-\beta_ix_{i-3}}{w_{i-2}};\ z_i=\frac{e_i-\alpha_ix_{i-2}-\beta_iy_{i-3}}{w_{i-1}};\\&w_i=a_i-z_ix_{i-1}-\alpha_iy_{i-2}-\beta_id_{i-3};\\&x_i=b_i-z_iy_{i-1}-\alpha_id_{i-2};\ y_i=c_i-z_id_{i-1};\\&m_i=h_i-z_im_{i-1}-\alpha_im_{i-2}-\beta_im_{i-3}\end{aligned} \tag{S4.5}$$

These calculations enable the computation of the unknown concentrations using backward recursion, as appropriate:

$$\begin{aligned}&u_N=\frac{m_N}{w_N};\ u_{N-1}=\frac{m_{N-1}-x_{N-1}u_N}{w_{N-1}};\ u_{N-2}=\frac{m_{N-2}-x_{N-2}u_{N-1}-y_{N-2}u_N}{w_{N-2}}\\&\mathit{for}\ i=N-3\rightarrow 1,\\&u_i=\frac{m_i-x_iu_{i+1}-y_iu_{i+2}-d_iu_{i+3}}{w_i}\end{aligned} \tag{S4.6}$$

S5. EC' Reaction in a Very Thin Layer

We consider the EC' reaction in an ultrathin layer adjacent to the electrode surface – a case previously considered by Laviron [S1], Savéant [S2] and Matsuda [S7]. This model assumes that the scan rate and the thin film thickness are sufficiently small so that the concentrations across the film are uniform throughout the heterogeneous electron transfer process, which itself

is considered to be electrochemically reversible (occurs with fast electrode kinetics).

Defining the dimensionless overpotential as $\varepsilon = \frac{F}{RT}\left(E - E^{0'}\right)$ enables the Nernstian equilibrium between the redox forms of the mediator to be described by $c_Q = c_P e^{\varepsilon}$. Thus, when catalysis occus ($k_{EC'} \neq 0$),

$$\begin{aligned} \frac{dc_P}{dt} &= k_{EC'} e^{\varepsilon} c_P c_M \\ \frac{dc_M}{dt} &= -\frac{dc_P}{dt} \end{aligned} \tag{S5.1}$$

Since mass conservation requires that, at any time t, $c_P^0 = c_P + c_Q$, equations (S5.1) can be solved for the boundary condition case that when $\varepsilon = u = \frac{F}{RT}\left(E_i - E^{0'}\right)$, $c_M = c_M^0$, so that $c_M = c_M^0 \left\{\frac{1+e^u}{1+e^{\varepsilon}}\right\}^{\lambda}$, where E_i is the initial potential used and the kinetic parameter is given as in the main text, *viz.*, $\lambda = \frac{RT}{Fv} k_{EC'} c_P^0$. Hence, when catalysis takes place, the rate of change of the concentration of the reduced form of the mediator is:

$$\left(\frac{dc_P}{dt}\right)_{cat} = k_{EC'} \frac{\left(1+e^u\right)^{\lambda}}{\left(1+e^{\varepsilon}\right)^{1+\lambda}} e^{\varepsilon} c_P^0 c_M^0 \tag{S5.2}$$

The total current flowing (i) is the summation of that in the presence (i_{cat}) and absence (i_0) of the homogeneous chemical reaction:

$$i = i_0 + i_k \tag{S5.3}$$

with:

$$\begin{aligned} i_0 &= -FV_{layer} \frac{dc_P}{dt} \\ i_{cat} &= -FV_{layer} \left(\frac{dc_P}{dt}\right)_{cat} \end{aligned} \tag{S5.4}$$

in which V_{layer} refers to the volume of the very thin film. In the absence of catalysis, $i_0 = FV_{layer}\left\{\frac{Fv}{RT}c_P^0\frac{e^{\varepsilon}}{\left(1+e^{\varepsilon}\right)^2}\right\}$, so that equation (S5.4) can be written as:

$$i = \frac{F^2 V_{layer} v c_P^0}{RT}\frac{e^{\varepsilon}}{\left(1+e^{\varepsilon}\right)^2}\left\{1+\frac{RT}{Fv}k_{EC'}c_M^0\frac{\left(1+e^{u}\right)^{\lambda}}{\left(1+e^{\varepsilon}\right)^{\lambda-1}}\right\} \qquad \text{(S5.5)}$$

Note that when u is sufficiently negative, equation (S5.5) gives rise to the expression developed by Laviron [S1].

Using the expression for the dimensionless current, $\psi = \frac{i}{2\pi F D_P a c_P^0}$, where a is the radius of the basal contacting surface of the droplet, and recognising that the volume of a droplet under thin-layer conditions is $V_{layer} = \frac{1}{3}\pi a^3\frac{\sin\theta\left(2+\cos\theta\right)}{\left(1+\cos\theta\right)^2}$, equation (S5.5) can be reformulated as:

$$\psi = \frac{1}{6}s^2\frac{\sin\theta\left(2+\cos\theta\right)}{\left(1+\cos\theta\right)^2}\frac{e^{\varepsilon}}{\left(1+e^{\varepsilon}\right)^2}\left\{1+\lambda\gamma\frac{\left(1+e^{u}\right)^{\lambda}}{\left(1+e^{\varepsilon}\right)^{\lambda-1}}\right\} \qquad \text{(S5.6)}$$

S6. Mechanism for the Mediated Oxidation of H_2S or Sulphydryl Thiols

The mediated oxidation of H_2S or sulphydryl thiols is an attractive study, owing to the relevance of these species within the oil and gas industry [S28], in biomedicine [S29] and in analytical chemistry [30]. The reaction upholds all three of the criteria outlined by Feldberg and Campbell [S16], with the additional benefit that the substrate can diffuse faster than the oxidised or reduced forms of the mediators. The reaction is, however, competitive, since both the protonated and the deprotonated forms of the substrate can react. In equation (S6.1), we illustrate this for a general sulphydryl thiol, RSH:

$$
\begin{aligned}
&RSH \rightleftarrows RS^{-} + H^{+} \\
&P - e^{-} \rightleftarrows Q \\
&Q + RSH \xrightarrow{k_1} P + RSH^{\bullet +} \\
&Q + RS^{-} \xrightarrow{k_2} P + RS^{\bullet} \\
&RSH^{\bullet +} \xrightarrow{fast} RS^{\bullet} + H^{+} \\
&RS^{\bullet} \xrightarrow{fast} \frac{1}{2} RSSR
\end{aligned}
\qquad \text{(S6.1)}
$$

The simultaneous oxidations afford a general kinetic law given in equation (S6.2). This recognises that, prior to electrolysis, the concentrations of the protonated and deprotonated thiol exist in equilibrium, characterised by the acidity constant, $K_a = \frac{c_{RS^-} c_{H^+}}{c_{RSH}}$, and the mass balance: $c_{RSH,0} = c_{RSH} + c_{RS^-}$.

$$
\frac{dc_P}{dt} = k_1 c_Q c_{RSH} + k_2 c_Q c_{RS^-} = k_{eff} c_Q c_{RSH,0} \qquad \text{(S6.2)}
$$

in which the effective bimolecular rate constant is:

$$
k_{eff} = \frac{k_1 c_{H^+} + k_2 K_a}{K_a + c_{H^+}} \qquad \text{(S6.3)}
$$

Note that for the case of L-cysteine, the acidity constant refers to the third constant (K_{a3}), as outlined in the main paper.

S7. References

[1]. E. Laviron, The elucidation and study of the mechanism of chemical surface reactions using linear sweep voltammetry, *Chemie Ing. Techn.*, 1972, **44**, 183.

[2]. C. P. Andrieux, J.-M. Savéant, Heterogeneous (chemically modified electrodes, polymer electrodes) *vs.* homogeneous catalysis of electrochemical reactions, *J. Electroanal. Chem.*, 1978, **93**, 163.

[3]. J. Facci, R. W. Murray, Charge transport by electron exchange cross reaction in cyclic voltammetry of $IrCl_6^{3-}$–$Fe(CN)_6^{3-}$ mixtures trapped in polycationic films on electrodes, *J. Phys. Chem.*, 1981, **85**, 2870.

[4]. J.-M. Savéant, K. B. Su, Homogeneous redox catalysis of electrochemical reactions, VI: zone diagram representation of the kinetic regimes, *J. Electroanal. Chem.*, 1984, **171**, 341.

[5]. C. P. Andrieux, P. Hapiot, J.-M. Savéant, Electron transfer coupling of diffusional pathways: potential-step chronoamperometry and cyclic voltammetry of cobalt(II) tetraphenylporphyrin and 4-nitrotoluene in dimethylformamide solutions, *J. Electroanal. Chem.*, 1985, **186**, 237.

[6]. C. P. Andrieux, P. Hapiot, J.-M. Savéant, Electron transfer coupling of diffusional pathways: homogeneous redox catalysis of dioxygen reduction by the methylviologen cation radical in acidic dimethylsulfoxide, *J. Electroanal. Chem.*, 1985, **189**, 121.

[7]. K. Aoki, K. Tokuda, H. Matsuda, Linear sweep and cyclic voltammetry for electrocatalysis at modified electrodes with very thin films, *J. Electroanal. Chem.*, 1986, **199**, 69.
[8]. G. Denuault, M. Fleischmann, D. Pletcher, O. R. Tutty, Development of the theory for the interpretation of steady-state limiting currents at a microelectrode: EC' processes – first- and second-order reactions, *J. Electroanal. Chem.*, 1990, **280**, 243.
[9]. G. Taylor, H. H. Girault, J. McAleer, Digital simulation of charge transfer to an ultramicrodisc interface, *J. Electroanal. Chem.*, 1990, **293**, 19.
[10]. R. G. Compton, A. C. Fisher, R. A. Spackman, Homogeneous catalysis of electrochemical reactions: channel electrode voltammetry and the EC' mechanism, *Electroanalysis*, 1992, **4**, 167.
[11]. A. Gennaro, A. A. Isse, J.-M. Savéant, M.-G. Severin, E. Vianello, Homogeneous electron transfer catalysis of the electrochemical reduction of carbon dioxide: do anion radicals react in an outer-sphere manner? *J. Am. Chem. Soc.*, 1996, **118**, 7190.
[12]. Y. Xie, C. Kang, F. C. Anson, Calculation of cyclic voltammetric responses for the reductive formation of catalyst-substrate adducts on electrode surfaces, *J. Chem. Soc., Faraday Trans.*, 1996, **92**, 3917.
[13]. J. J. Pietron, R. W. Murray, Mediated electrocatalysis with polyanthraquinone-functionalised monolayer-protected clusters, *J. Phys. Chem. B*, 1999, **103**, 4440.
[14]. S. C. B. Abercrombie, G. Denuault, Steady-state simulation of electrode processes with a new error-bounded adaptive finite element algorithm, *Electrochem. Commun.*, 2003, **5**, 647.
[15]. R. Senthamarai, L. Rajendran, A comparison of diffusion-limited currents at microelectrodes of various geometries for EC' reactions, *Electrochim. Acta*, 2008, **53**, 3566.
[16]. S. W. Feldberg, J. F. Campbell, The quasi-catalytic mechanism: a variation of the catalytic (EC') mechanism, *Anal. Chem.*, 2009, **81**, 8797.
[17]. E. S. Rountree, B. D. McCarthy, T. T. Eisenhart, J. L. Dempsey, Evaluation of homogeneous electrocatalysts by cyclic voltammetry, *Inorg. Chem.*, 2014, **53**, 9983.
[18]. K. R. Ward, N. S. Lawrence, R. S. Hartshorne, R. G. Compton, Cyclic voltammetry of the EC' mechanism at hemispherical particles and their arrays: the split wave, *J. Phys. Chem. C*, 2011, **115**, 11204.
[19]. D. J. Martin, B. D. McCarthy, E. S. Rountree, J. L. Dempsey, Qualitative extension of the EC' zone diagram to a molecular catalyst for a multi-electron, multi-substrate electrochemical reaction, *Dalton Trans.*, 2016, **45**, 9970.
[20]. C. Costentin, D. G. Nocera, C. N. Brodsky, Multi-electron, multi-substrate molecular catalysis of electrochemical reactions: formal kinetic analysis in the total catalysis regime, *Proc. Natl. Acad. Sci.* U.S.A., 2017, **114**, 11303.
[21]. C. Costentin, J.-M. Savéant, Homogeneous molecular catalysis of electrochemical reactions: manipulating intrinsic and operational factors for catalyst improvement, *J. Am. Chem. Soc.*, 2018, **140**, 16669.
[22]. N. S. Lawrence, M. Thompson, C. Prado, L. Jiang, T. G. J. Jones, R. G. Compton, Amperometric detection of sulphide at a boron-doped diamond electrode: the electrocatalytic reaction of sulphide with ferricyanide in aqueous solution, *Electroanalysis*, 2002, **14**, 499.
[23]. T. S. Varley, N. S. Lawrence, J. D. Wadhawan, Electrochemical goniometry: keystone reactivity at the three-phase boundary, *J. Solid State Electrochem.*, 2025, **29**, 3.
[24]. C. Amatore, A. Oleinick, I. Svir, Diffusion within nanometric and micrometric spherical-type domains limited by nanometric ring or pore active interfaces: Part I – conformal mapping approach, *J. Electroanal. Chem.*, 2005, **575**, 103.
[25]. H. Matsuda, Y. Ayabe, Zúr theorie der Randles-Sevčíkschen kathodenstrahl-polarographie, *Z. Elektrochem.*, 1955, **59**, 494.
[26]. H. J. Ward, R. A. Ward, N. S. Lawrence, J. D. Wadhawan, Unravelling the occurrence of mediator-blood protein interactions *via* the redox catalysis of the physiological gasotransmitter hydrogen sulphide, *ChemistrySelect*, 2021, **6**, 10059.

[27]. O. V. Klymenko, I. Svir, A. Oleinick, C. Amatore, A novel approach to the simulation of electrochemical mechanisms involving acute reaction fronts at disc and band microelectrodes, *ChemElectroChem*, 2012, **13**, 845.
[28]. R. A. Marriott, P. Pirzadeh, J. J.Marrugo-Hernandez, S. Ravel, Hydrogen sulphide formation in oil and gas, *Can. J. Chem.*, 2016, **94**, 406.
[29]. B. L. Predmore, D. J. Lefer, G. Gojon, Hydrogen sulphide in biochemistry and medicine, *Antioxid. Redox Signal.*, 2012, **17**, 119.
[30]. P. C. White, N. S. Lawrence, J. Davis, R. G. Compton, Electrochemical determination of thiols: a perspective, *Electroanalysis*, 2002, **14**, 89.
