# Supplementary material for: Are redox catalytic reaction rates accelerated in microdroplets on electrode surfaces?
Source: J Solid State Electrochem. 2025 Apr 12;29(6):2321–34. doi: 10.1007/s10008-025-06283-4 (PMC12103339; doi:10.1007/s10008-025-06283-4)
Supplement: Supplementary file 3 — Supplementary file3 (PDF 189 KB) [file 10008_2025_6283_MOESM3_ESM.pdf]

RightsLink

Sign in/Register

Oxidation of Cysteine by Electrogenerated Hexacyanoferrate(III) in Microliter Droplets

Author: Kathryn J. Vannoy, Jeffrey E. Dick

Publication: Langmuir

Publisher: American Chemical Society

Date: Oct 1, 2022

Copyright © 2022, American Chemical Society

PERMISSION/LICENSE IS GRANTED FOR YOUR ORDER AT NO CHARGE

This type of permission/license, instead of the standard Terms and Conditions, is sent to you because no fee is being charged for your order. Please note the following:

- Permission is granted for your request in both print and electronic formats, and translations.
- If figures and/or tables were requested, they may be adapted or used in part.
- Please print this page for your records and send a copy of it to your publisher/graduate school.
- Appropriate credit for the requested material should be given as follows: "Reprinted (adapted) with permission from {COMPLETE REFERENCE CITATION}. Copyright {YEAR} American Chemical Society." Insert appropriate information in place of the capitalized words.
- One-time permission is granted only for the use specified in your RightsLink request. No additional uses are granted (such as derivative works or other editions). For any uses, please submit a new request.

If credit is given to another source for the material you requested from RightsLink, permission must be obtained from that source.

BACK CLOSE WINDOW

© 2025 Copyright - All Rights Reserved | Copyright Clearance Center, Inc. | Privacy statement | Data Security and Privacy | For California Residents | Terms and ConditionsComments? We would like to hear from you. E-mail us at customercare@copyright.com

Privacy - Terms
